# Staphylococcus caseorum sp. nov., a new species isolated from Spanish traditional, blue-veined Cabrales cheese

**DOI:** 10.1099/ijsem.0.007144

**Published:** 2026-04-27

**Authors:** Lucía Vázquez, Javier Rodríguez, Ana Belén Flórez, Baltasar Mayo

**Affiliations:** 1 Departamento de Microbiología y Bioquímica, Instituto de Productos Lácteos de Asturias (IPLA), Consejo Superior de Investigaciones Científicas (CSIC), Francisco Pintado Fe, 26, 33011-Oviedo, Spain; 2 Instituto de Investigación Sanitaria del Principado de Asturias (ISPA), Avenida de Roma s/n, 33011-Oviedo, Spain

**Keywords:** blue-veined cheese, Cabrales, cheese, cheese microbiota, coagulase-negative staphylococci, *Staphylococcus*, starters

## Abstract

Three bacterial strains isolated from Cabrales cheese (IPLA 37010, IPLA 37011 and IPLA 37012^T^) were initially assigned to *Staphylococcus equorum* based on partial 16S rRNA gene sequencing. Nevertheless, genome comparison with the type strains *S. equorum* subsp. *equorum* DSM 20674^T^ and *S. equorum* subsp. *linens* DSM 15097^T^ revealed them to possess distinct genomic and genotypic features. They were therefore subjected to a series of extensive phenotypic tests, including carbohydrate fermentation, enzyme activity, novobiocin resistance, growth in aerobiosis and anaerobiosis and at different pH, temperature and salt concentrations. All three strains were found to be facultatively anaerobic, coagulase- and oxidase-negative and catalase-positive. Matrix-assisted laser desorption/ionization time-of-flight analysis was unable to assign the strains to any staphylococcal species with validly published names. Based on these genomic, phenotypic and chemotaxonomic results, IPLA 37010, IPLA 37011 and IPLA 37012^T^ are concluded to represent a novel species, for which the name *Staphylococcus caseorum* sp. nov. is proposed. The type strain, *S. caseorum* IPLA 37012^T^, is deposited in the German Collection of Microorganisms and Cell Cultures (DSM 117647^T^) and in the Spanish Type Culture Collection (CECT 31035^T^).

## Introduction

Staphylococci are widely distributed bacteria found in diverse environments, including foods, particularly fermented foods [1,2], and as common members of the commensal microbiota of humans and animals [3]. At the time of writing, the genus *Staphylococcus* comprised 78 species with validly published names and 30 subspecies according to the List of Prokaryotic names with Standing in Nomenclature (https://lpsn.dsmz.de//; accessed on 19 February 2026). Based on their ability to produce the enzyme coagulase, staphylococci are classified as either coagulase-positive (CoPS) or coagulase-negative (CoNS). CoPS are opportunistic pathogens in humans and animals. They carry a large number of virulence factors and possess various mechanisms that afford them resistance to antibiotics [4]. With some exceptions, of which *Staphylococcus epidermidis*, *Staphylococcus haemolyticus* and *Staphylococcus lugdunensis* are the most clinically relevant species [5], CoNS species are mostly considered as commensal and beneficial bacteria [3]. In fermented foods, they produce proteases and lipases that lead to the formation of lower molecular weight compounds (such as amino acids, fatty acids, esters and aldehydes), which may play a role in the development of flavour, aroma and texture [2,6]. CoNS are also responsible for the reduction of nitrate to nitrite in fermented meat, again enhancing aroma and preserving colour [6]. Owing to these properties, strains of several CoNS species, including *Staphylococcus carnosus*, *Staphylococcus succinus*, *Staphylococcus xylosus* and *Staphylococcus equorum*, have been proposed and even used as ripening agents in food fermentations [2,7]. CoNS species may also contribute to food safety and quality by inhibiting the growth of pathogens and spoilage micro-organisms [8,9].

Cabrales cheese is one of the most prestigious traditional, blue-veined cheeses produced in Spain. It has enjoyed protected designation of origin (PDO) status since 1981. According to the PDO Technical Annex [10], Cabrales cheese is made with raw cow’s milk or a mixture of cow’s, sheep’s and goat’s milk. Its production is restricted to a few municipalities in the Principality of Asturias in northern Spain, where its ripening is required to occur in natural karstic caves [10]. Traditionally, the acidification of the curd relied on the native microbiota of the milk and micro-organisms from the production and ripening environments [11]. Nowadays, however, the addition of acidifying starters (lactic acid bacteria) and ripening cultures (*Penicillium roqueforti*) is a common practice that ensures standardized production and consistent quality. Pioneering culturing studies revealed the wide diversity of prokaryotic and eukaryotic micro-organisms in this cheese [12,13], with *Lactococcus lactis* as the dominant species throughout manufacturing and ripening. The recent use of next-generation sequencing techniques has, however, revealed a microbial diversity even larger than that anticipated by culturing techniques [14,15]. Indeed, a myriad of previously unreported taxa have been identified, including species of atypical genera in cheese, such as *Atopostipes*, *Alkalibacterium*, *Marinobacter*, *Mesonia*, *Oceanisphera*, *Bavaricoccus* and *Yaniella*, among others. These methods have also shown species of *Lactococcus*, *Tetragenococcus* and *Staphylococcus* to be the majority populations among the bacterial contingent. In a recent study [16], *S. equorum* strains have been recovered and their antibiotic resistance/susceptibility profiles determined. Intriguingly, three strains showed digital DNA–DNA hybridization (dDDH) and ortho-average nucleotide identity (ortho-ANI) values below the established thresholds for species delineation, suggesting they may be representatives of a new taxon.

This work reports the phenotypic, genetic, genomic and chemotaxonomic characterization of three *S. equorum*-like strains (IPLA 37010, IPLA 37011 and IPLA 37012^T^) and a comparison of their properties with those of the type strains *S. equorum* subsp. *equorum* DSM 20674^T^ and *S. equorum* subsp. *linens* DSM 15097^T^. In view of the results, it is proposed that these strains constitute a novel *Staphylococcus* species, for which the name *Staphylococcus caseorum* sp. nov. is proposed.

## Methods

### Isolation and culture conditions

Three strains – IPLA 37010 (5A3I), IPLA 37011 (11A1I) and IPLA 37012^T^ (30A2I) – had been isolated in a previous work from Cabrales cheese samples and assigned to *S. equorum* by 16S rRNA gene amplification, sequencing and sequence comparison [16]. The type strains *S. equorum* subsp. *equorum* DSM 20674^T^ and *S. equorum* subsp. *linens* DSM 15097^T^ were purchased from the German Collection of Microorganisms and Cell Cultures (DSMZ; Braunschweig, Germany). *Staphylococcus aureus* subsp. *aureus* RN4220 and *Limosilactobacillus reuteri* LR32 were obtained from the IPLA laboratory collection. Unless otherwise indicated for the different tests, staphylococcal strains were routinely cultured in brain heart infusion (BHI) broth or agar (VWR International, Radnor, PA, USA) supplemented with 5% (w/v) NaCl. Additionally, strains were grown in *Staphylococcus* Medium 110 (Oxoid, Basingstoke, UK), a selective and differential medium for staphylococci. *L. reuteri* LR32 was grown at 37 °C in MRS (Oxoid).

### Phylogenetic analysis

Total genomic DNA from IPLA 37010, IPLA 37011 and IPLA 37012^T^ was isolated from overnight cultures using the QIAamp DNA Mini Kit (Qiagen, Hilden, Germany) in accordance with the manufacturer’s protocol, with the modification of supplementing the lysis step with 5 µl of lysostaphin (2 mg ml⁻¹). The quality and purity of the purified genomic DNA were measured spectrophotometrically and quantity assessed with a Qubit fluorometer (ThermoFisher Scientific, Waltham, MA, USA) using the dsDNA Broad Range Assay Kit (ThermoFisher Scientific). To cover the complete 16S rRNA gene, different segments were amplified from total genomic DNA using the universal prokaryotic primers 27F (5′-AGAGTTTGATCCTGGCTCAG-3′), 1492R (5′-GGTTACCTTGTTACGACTT-3′) [17], Y1 (5′-TGGCTCAGGACGAACGCTGGCGGC-3′) and Y2 (5′-CCTACTGCTGCCTCCCGTAGGAGT-3′) [18] and 968F (5′-AACGCGAAGAACCTTAC-3′) [19], employing the PCR conditions reported by the above authors. Amplicons were purified using GenElute PCR Clean-Up columns (Sigma-Aldrich, St. Louis, MO, USA) and sequenced with the above primers by the Sanger method at Macrogen (Madrid, Spain). Nearly full-length 16S rRNA sequences were generated using Vector NTI Advance v.11.5 software (ThermoFisher Scientific).

Phylogenetic analyses were also performed involving the routinely used housekeeping genes *aroE*, *atpA*, *ddlA*, *dnaE*, *dnaJ*, *glpF*, *gmk*, *groEL*, *mutS*, *gapA*, *gapN*, *pyrE*, *pheS*, *pta*, *recN*, *rpoA*, *rpoB*, *sodA*, *thr*S and *tuf*. These genes are deemed appropriate for separating *Staphylococcus* species [20,21]. The respective sequences for the cheese strains were retrieved from their assembled genomes, while those for *S. equorum* subsp. *linens* DSM 15097^T^, *S. equorum* subsp. *equorum* DSM 20674^T^, *Staphylococcus saprophyticus* ATCC 15305^T^, *S. succinus* DSM 14617^T^ and *S. xylosus* CCM 2738^T^ were retrieved from genomes downloaded from the National Center for Biotechnology Information (NCBI) Assembly resource and annotated database using the Bacterial and Viral Bioinformatics Resource Center’s (BV-BRC) annotation system (v.3.46.3) using the rapid annotation using subsystems technology (RAST) tool kit (RASTtk) (https://www.bv-brc.org). Alignment analyses were then performed using the Molecular Evolutionary Genetics Analysis (MEGA) software v.11.0 [22], employing the muscle algorithm for alignment. Phylogenetic distances were inferred using the maximum likelihood method based on the general time reversible model with a bootstrap test of 1,000 replications.

### Genomic and phylogenomic analyses

The genome sequence of IPLA 37010, IPLA 37011 and IPLA 37012^T^ strains using short-read Illumina technology has already been analysed and reported [16]. In the present work, the genomes of the three strains were isolated and sequenced using long-read Pacific Bioscience (PacBio) whole-genome sequencing (WGS) technology. For this, total genomic DNA isolated and purified as above was sequenced in a PacBio RS II instrument (Pacific Biosciences, Menlo Park, CA, USA) at FISABIO Sequencing and Bioinformatics Service (FISABIO, Valencia, Spain). A hybrid genome assembly of short- and long-read sequences was generated using Unicycler v.0.4.8 software [23] and polished using Racon v.1.4.20 [24] and Pilon v.1.23 [25] software.

WGS data were analysed following the minimal standards for the delineation of new staphylococcal species [26], the recent recommendations of WGS analysis for delineating new bacterial species [27], and in compliance with Recommendation 30 of the International Code of Nomenclature of Prokaryotes [28]. Genomic features were annotated in the BV-BRC annotation system using RASTtk. Genome sequences were also inspected using several bioinformatic programmes. Additionally, clustered regularly interspaced short palindromic repeats (CRISPR)-Cas (CRISPR associated) nuclease systems were sought using the CRISPRCasTyper v.1.8.0 tool (https://crisprcastyper.crispr.dk; accessed on 16 February 2025), and prophages were predicted using the PHAge Search Tool with Enhanced Sequence Translation (https://phastest.ca/; accessed on 16 February 2025). Ribosomally synthesized and post-translationally modified peptides and bacteriocins were searched for using BAGEL4 (bagel4.molgenrug.nl/) (accessed on 16 February 2025). Finally, virulence and pathogenicity factors in the genomes were searched against the virulence factor database (VFDB; http://www.mgc.ac.cn/VFs/) and victors database (https://phidias.us/victors/). Comparative genome analysis between cheese strains and *S. equorum* type strains was carried out by using an in-house-developed Python-based algorithm and visualized with Matplotlib (https://matplotlib.org/).

The ortho-ANI [29] and dDDH [30] values were calculated using the ANI calculator tool from the EZBioCloud (https://www.ezbiocloud.net/tools/ani) and the type (strain) genome server (TYGS) platform (https://tygs.dsmz.de/) [31] of the DSMZ, respectively. Phylogenomic analyses of hybrid-assembled genome sequences of short and long reads were done at the TYGS platform using the genome-to-genome distance calculator 4.0 tool (http://ggdc.dsmz.de/). A phylogenomic tree of staphylococcal cheese strains and type strains of the closest *Staphylococcus* species was inferred by genome blast distance phylogeny (GBDP) with FastME 2.1.6.1 at the DSMZ Type Strain Genome Server (http://ggdc.dsmz.de/).

### Morphology, physiology and biochemical characteristics

Cell morphology was examined using a Leica DMi8 inverted optical microscope (Leica, Wetzlar, Germany). Bacterial cells were collected at exponential phase, concentrated to 1:10 in filtered fresh medium and immobilized in low melt agarose pads before photographing under bright field illumination using a Leica DFC 365 FX camera. The length of the diameter of ten bacterial cells per strain was measured using the LAS X software platform (Leica). Detailed examination of cell morphology was also performed using a Jeol 1400 transmission electron microscope (TEM) with a Gatan ES1000Ww camera (Jeol, Peabody, MA, USA). For this, cells were collected at exponential phase and fixed immediately in 4% paraformaldehyde (Electron Microscopy Sciences, Hatfield, PA, USA) plus 5% glutaraldehyde (ThermoFisher Scientific) in 1×PBS for 10 min at room temperature. Cells were mixed into the buffered fixative solution and left at room temperature for 2 h. Samples were then washed with 1×PBS, post-fixed (1 h, 4 °C) with 1% osmium tetroxide (TAAB Laboratories, Aldermaston, UK) in 0.8% potassium ferricyanide (Sigma-Aldrich), and incubated with 2% aqueous uranyl acetate (Electron Microscopy Sciences) for 40 min at 4 °C. After washing with distilled water, samples were dehydrated with increasing concentrations of anhydrous acetone (VWR International, Llinars del Vallés, Spain) and embedded in TAAB 812 epoxy resin (TAAB Laboratories). Polymerization was carried out in 100% epoxy resin at 60 °C for 2 days. Resin blocks were trimmed, and ultrathin 70 nm-thick sections obtained with a UC6 ultramicrotome (Leica) were transferred to 400 mesh nickel grids (Gilder Grids, Grantham, UK) before staining with 2% aqueous uranyl acetate for 15 min and 0.2% lead citrate (Electron Microscopy Sciences) for 1 min at room temperature. Sections were visualized using a Jeol JEM 1400 Flash electron microscope operating at 100 kV. Micrographs were taken with a Gatan OneView digital camera (Jeol) at various magnifications.

The ability to bind fibrinogen and form macroscopic aggregates (clumping factor) was assessed with the Pastorex *Staph* Plus kit (Bio-Rad, Hercules, CA, USA), following the supplierʼs recommendations. Catalase activity was assessed by the slide catalase test with hydrogen peroxide (3%). Oxidase activity was determined using the filter paper spot method employing Kovacsʼ oxidase reagent (Sigma-Aldrich) at 1%. DNase (nuclease) activity was assayed with intact cells and heat-inactivated cell-free extracts on blue toluidine DNase agar (Oxoid) following the protocol by Yu *et al.* [32]. Haemolytic activity was examined by streaking a colony on tryptone soy agar plates supplemented with 5% horse or sheep blood (Oxoid). Plates were incubated for 24 h at 37 °C and then stored at 4 °C for 12 h to reveal *α*- and *β*-haemolysis. To assess *δ*-haemolysis, strains were cross-streaked perpendicularly with *S. aureus* subsp. *aureus* RN4220, a *β*-haemolysin producer.

Together with the type strains of the two *S. equorum* subspecies, cheese strains were subjected to an extensive phenotypic characterization using the commercial API Staph ID 32, API 50 CH and API ZYM systems (bioMérieux, Marcy-l'Étoile, France) following the supplierʼs recommendations and after incubation at 32 °C. The production of biogenic amines was evaluated by incubating the strains in BHI supplemented with 1 mM of the precursor substrate amino acids (histidine, tyrosine and ornithine) and, after growth, searching for them by ultra HPLC analysis as previously reported [33]. *L. reuteri* LR32, a tyramine and histamine producer [34], was grown under its optimal growth conditions and used as a positive control. The MIC of novobiocin to all three cheese strains was tested using a standardized broth microdilution method, as previously reported [35]. The test was performed in duplicate in Mueller-Hinton broth (Oxoid) using ELISA plates (ThermoFisher Scientific) containing twofold dilutions of the antibiotic.

Growth in anaerobiosis was tested at 32 °C in BHI incubated in an anaerobic jar with Anaerocult A (Merck, Darmstadt, Germany). The effect of temperature, pH and salt content on growth was evaluated after cultivation on BHI agar plates supplemented with 2.5% (w/v) NaCl (3% in total) at 32 °C for 48 h. Temperature tolerance was evaluated at 5 and 10 °C (simulating cheese maturation conditions), 20, 32 and 37 °C (both static and with shaking) and at 42 °C. pH tolerance was evaluated at pH 7.4 (basic media pH) and across a range from 7.0 down to 4.5 in 0.25 intervals. Tolerance to salinity was determined using BHI medium supplemented with NaCl concentrations of 0.5% (basal medium), 2.5, 5, 10, 15, 20 and 25%. Cultured cells were suspended in 0.9% NaCl and adjusted to a density of McFarland standard 1. Suspensions were inoculated at 1% into fresh medium, modified as required for the conditions to be tested.

The behaviour of the strains in milk was tested in ultrahigh temperature (UHT) treated, semi-skimmed milk (CAPSA, Siero, Spain) supplemented with 0.5 g tryptone (Oxoid), incubated at 32 °C for 48 h. Production and/or consumption of sugars and organic acids during growth in milk were therefore sought by HPLC, as previously described [36]. In short, 5 ml of milk cultures was mixed with 25 ml of 5.4 mM H_2_SO_4_, and the mixture was shaken for 1 h at 37 °C before centrifuging at 4,500 ***g*** for 10 min. The supernatants were filtered through a 0.45 µm membrane and stored frozen until analysis. Organic acids were identified using a 996 Photodiode Array Detector (Waters, Milford, MA, USA) at 210 nm and sugars using a 410 Differential Refractometer (Waters) at 280 nm. Metabolite quantification was determined using calibration curves prepared with standards. Experiments were performed in duplicate.

Matrix-assisted laser desorption/ionization time-of-flight (MALDI-TOF) MS analysis of the present strains was performed in a BioTyper device (Bruker, Billerica, MA, USA) at the DSMZ service. The cell-wall peptidoglycan structure of the cheese strains was also determined at the DSMZ facilities under standardized conditions [37]. Amino acid identification and quantification were performed in total cell hydrolysates of the strains (100 °C, 4 N HCl, 16 h) by GC/MS analysis of *N*-heptafluorobutyric amino acid isobutyl esters.

## Results and discussion

### Isolation and identification

Three strains IPLA 37010 (5A3I), IPLA 37011 (11A1I) and IPLA 37012^T^ (30A2I) were isolated from a ripened Cabrales cheese (6–8 months old) in Sotres, a village in the Picos de Europa National Park (Principality of Asturias, northern Spain; approximate geographic coordinates of the manufacturing location and ripening cave 43° 13′ 58″ N 4° 44′ 55″ W, altitude 1,023 m) in October 2020. The three strains were made publicly available via the Spanish culture collection (CECT) under accession numbers CECT 31033 (IPLA 37010), CECT 31034 (IPLA 37011) and CECT 31035^T^ (IPLA 37012^T^) and via the DSMZ under accession numbers DSM 117647^T^ (IPLA 37012^T^), DSM 117648 (IPLA 37010) and DSM 117649 (IPLA 37011).

**Fig. 1.**
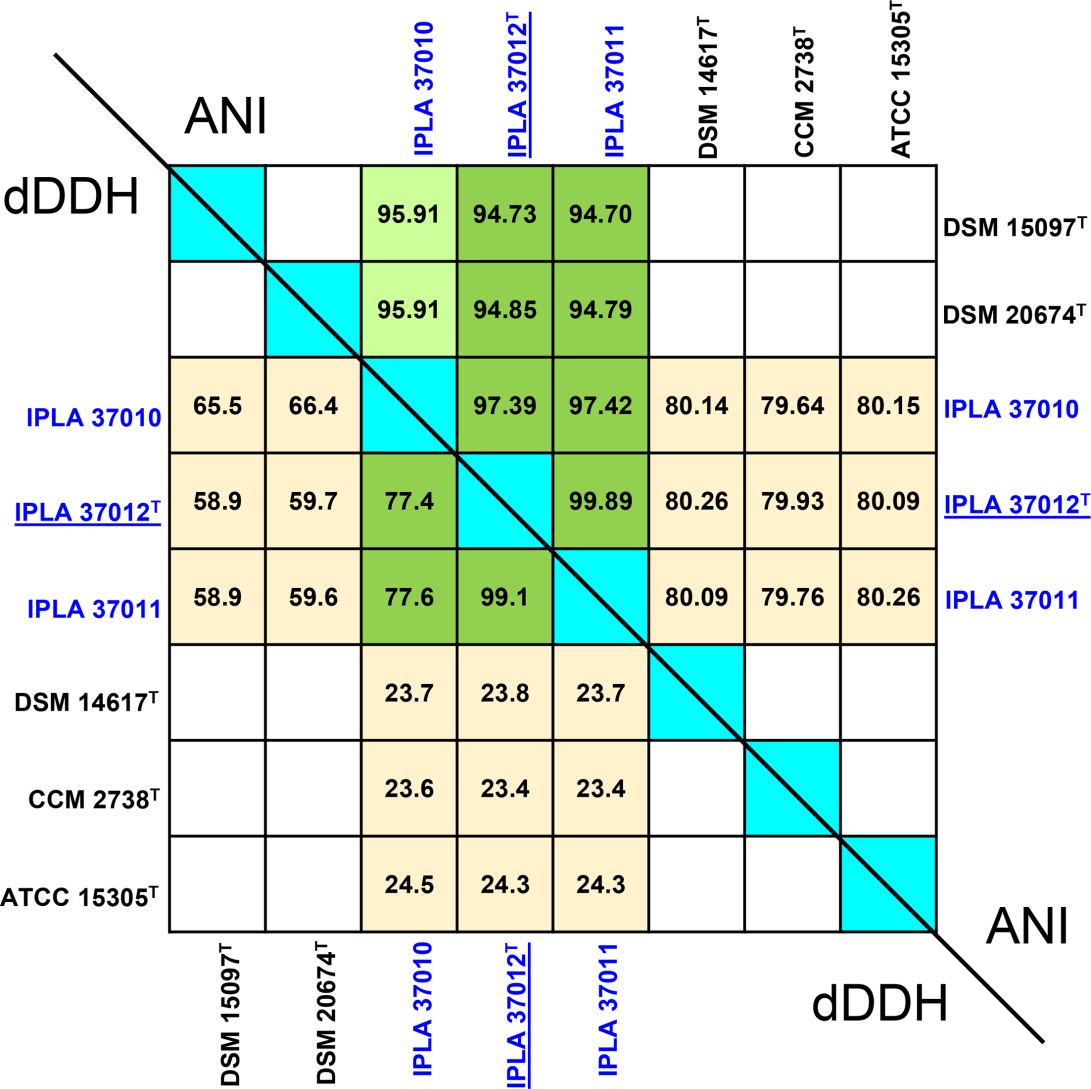
Genomic similarity of several *Staphylococcus* species type strains with the strains of the newly proposed *S. caseorum* species of this study. Results from dDDH are shown below the self-comparison diagonal, and results from ortho-ANI are shown above the diagonal. For comparative purposes, the genome of the type strains *S. equorum* subsp. *equorum* DSM 20674^T^, *S. equorum* subsp. *linens* DSM 15097^T^, *S. succinus* DSM 14617^T^, *S. xylosus* CCM 2738^T^ and *Staphylococcus saprophyticus* ATCC 15305^T^ was also included.

Although initially identified as *S. equorum* by 16S rRNA amplification and sequencing, genome sequence analysis of short-read sequencing of all three strains returned dDDH and ortho-ANI values within the range of 58.8–66.0% and 94.74–95.96%, respectively, with respect to the type strains *S. equorum* subsp. *equorum* DSM 20674^T^ and *S. equorum* subsp. *linens* DSM 15097^T^ [16], the current standard parameters for delineating species [29,30], suggesting they might belong to a different taxonomic unit. After genome sequencing performed in this work by long-read technology, the genome sequences were used again to calculate new dDDH and ortho-ANI values (Fig. 1). The dDDH values of the strains IPLA 37010, IPLA 37011 and IPLA 37012^T^ to the type strains of *S. equorum* subsp. *equorum* (DSM 20674^T^) and *S. equorum* subsp. *linens* (DSM 15097^T^) ranged from 58.9 to 66.4%, below the typical threshold for assigning strains to the same species (70%) [29,38], while the ortho-ANI values were on the verge of the species delineation threshold (94–96%) [30].

**Fig. 2.**
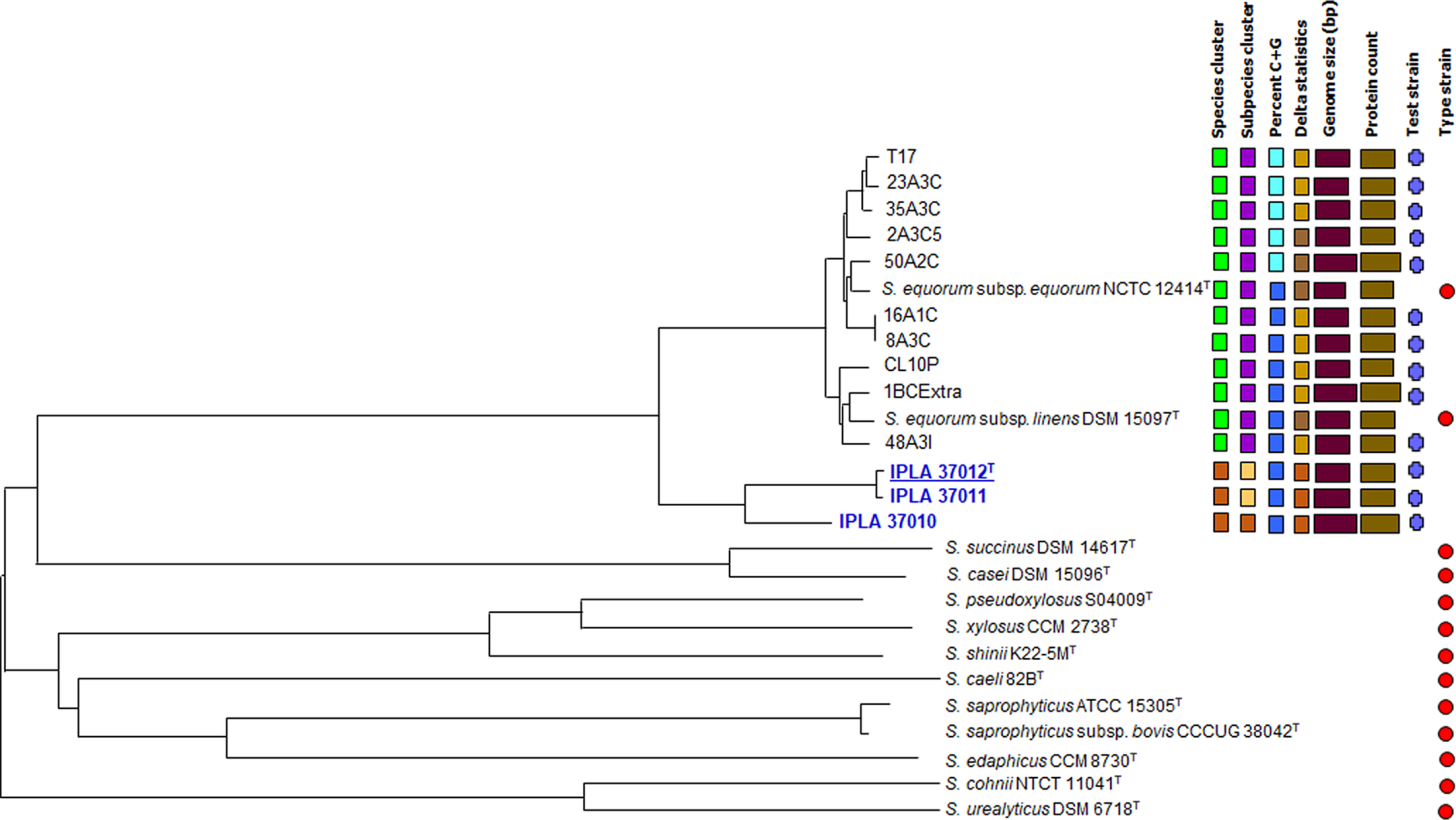
Phylogenomic analysis of the *Staphylococcus* spp. strains from cheese with the type strains of several staphylococcal species, as inferred from GBDP at the DSMZ TYGS (http://ggdc.dsmz.de/). In blue, the strains being proposed as a new species (underlined for the type strain). The tree was inferred. Branch lengths were scaled in terms of the approach distance of formula d5. The numbers above branches are GBDP pseudo-bootstrap support values >60% from 100 replications (average branch support of 85.2%). The scheme on the right denotes, with the same-coloured tone, strains considered as belonging to the same taxon.

Phylogenomic analyses of hybrid-assembled genome sequences of short- and long-read technologies showed IPLA 37010, IPLA 37011 and IPLA 37012^T^ to be well separated from the type strains of all *Staphylococcus* species, as well as from all other cheese isolates that had been assigned earlier [16] to either *S. equorum* subsp. *equorum* or *S. equorum* subsp. *linens* subspecies (Fig. 2). As shown in the figure, the strains of this study clustered together and branched from all others at the species level, which supported the idea that the three examined strains are members of a novel species taxon.

### Genomic features

The hybrid genome assembly of IPLA 37010 and IPLA 37012^T^ strains produced seven and six contigs, respectively. PCR amplification and sequencing of the amplicons using primers based on the extremes of the assembled sequences proved all contigs to be circular molecules corresponding to the bacterial chromosome plus six and five extrachromosomal plasmids for IPLA 37010 and IPLA 37012^T^, respectively. Congruently, the genomes of these two strains were considered to be complete. The hybrid assembly of short and long reads of IPLA 37011 produced 14 contigs that could not be further assembled; of these, 7 contigs were circular and may correspond to plasmids. The genome of this strain remains as a draft. Among the large extrachromosomal complement of the strains, the detection of a large plasmid of around 90 kbp in every strain is worthy of note. The genome sequences of all three strains were deposited at GenBank (BioProject accession number PRJNA1253107) under Genome Assembly accession numbers ASM4994321v1 (IPLA 37010), ASM4994313v1 (IPLA 37011) and ASM4994319v1 (IPLA 37012^T^).

The general properties of the draft genomes of IPLA 37010, IPLA 37011 and IPLA 37012^T^ are summarized in Table 1. The genome size ranged between 3.02 and 3.17 Mbp, a little larger than that reported for six *S. equorum* strains [39] and certainly bigger than those of the type strains *S. equorum* subsp. *equorum* DSM 20674^T^ (2.74 Mbp) and *S. equorum* subsp. *linens* DSM 15097^T^ (2.77 Mbp). At 32.97–33.01 mol%, the G+C content of the novel species is similar to that of DSM 20674^T^ and DSM 15097^T^ and within the expected range for *Staphylococcus* species [3]. As regards the large extrachromosomal complement harboured by all three strains (5–7 plasmids), a similar number of plasmids has been reported by the American Type Culture Collection (http://atcc.org/) in the genome of *S. equorum* subsp. *equorum* DSM 20674^T^ (from 2.2 to 28.6 kbp) and in strains from fermented seafood [40]. Annotation of the hybrid genome assemblies of IPLA 37010, IPLA 37011 and IPLA 37012^T^ showed 3,171, 3,058 and 2,976 coding sequences (CDSs); 60, 62 and 61 tRNA genes; and 7 copies each of the ribosomal operons (23S, 16S and 5S genes), plus an additional copy of the gene encoding the 5S rRNA molecule. Comparative genome analysis proved the cheese strains to contain a similar gene content and ribosomal operons to those of the two *S. equorum* subspecies type strains, *S. equorum* subsp. *equorum* DSM 20674^T^ (Genome assembly ASM2902496v1) and *S. equorum* subsp. *linens* DSM 15097^T^ (Genome assembly ASM290195v1). This analysis further showed a majority of the CDSs (2,159) to be orthologous genes shared by all five strains (Fig. S1, available in the online Supplementary Material). Although this, the number of *S. equorum* unique genes further suggested a certain evolutionary separation between *S. caseorum* and *S. equorum* taxa, which comprised 1,151 *S*. *caseorum*-specific CDSs. Of these, 443 were unique genes to IPLA 37010, 122 to IPLA 37011 and 81 to IPLA 37012^T^.

**Table 1.** General features of WGS of the cheese strains from the new proposed species *S. caseorum*

Feature/property	Strain		
	IPLA 37010	IPLA 37011	IPLA 37012**^T^**
**Assembly details**			
Genome size (bp)	3,172,315	3,072,447	3,025,197
G+C content (mol%)	33.01	32.97	33.10
No. of contigs	7	14	6
Chromosome (bp)	3,015,900	7 Contigs (1,881,025; 847,666; 123,854; 38,289; 3,251; 1,744; 931)	2,915,285
Plasmids (bp)	P1 (94,228); P2 (44,083); P3 (8,242); P4 (5,633); P5 (2,155); P6 (2,074)	P1 (96,128); P2 (45,359); P3 (14,419); P4 (11,345); P5 (3,450); P6 (2,392); P7 (2,594)	P1 (88,597); P2 (9,512); P3 (7,909); P4 (2,486); P5 (1,408)
**Annotated genome features**			
No. of CDSs	3,171	3,058	2,976
tRNA/rRNA	60/22	62/12*	61/22
Repeat regions	119	–*	83
Proteins with functional assignment	2,370	2,294	2,255
Hypothetical proteins	801	764	721
**Special features**			
Transporters (TCDB)	20	19	22
CRISPR loci array	1	–	–
CRISPR repeats/spacers	6/5	–	–
Bacteriocins (% of identity; homologous protein)	–	Lactococcin 972 (41; GBX02733.1)	Lactococcin 972 (41; GBX02733.1)
Predicted prophages	3 intact (34.6; 45.4; 47 kb); 1 questionable (19 kb); 5 incomplete (25.6 kb)	3 intact (34; 46.1; 51.9 kb)	2 intact (51.8; 65.9 kb); 1 questionable (34 kb)

*The small number of rRNA genes and the absence of repeat regions may result from incomplete assembly.

Analyses for virulence and pathogenicity factors in the VFDB revealed only ten hits (*oppD*, *pyrAA*, *mgrA*, *citB*, *clpX*, *recA*, *purL*, *SA1453*, *femB* and *trpB*). All these are housekeeping genes involved in cell metabolism, which suggests the strains of this study can be considered safe members of the commensal cheese microbiota. Further, the number of hits is considerably smaller than that found in pathogenic staphylococcal species [41,42].

### Phylogenetic analyses

**Fig. 3.**
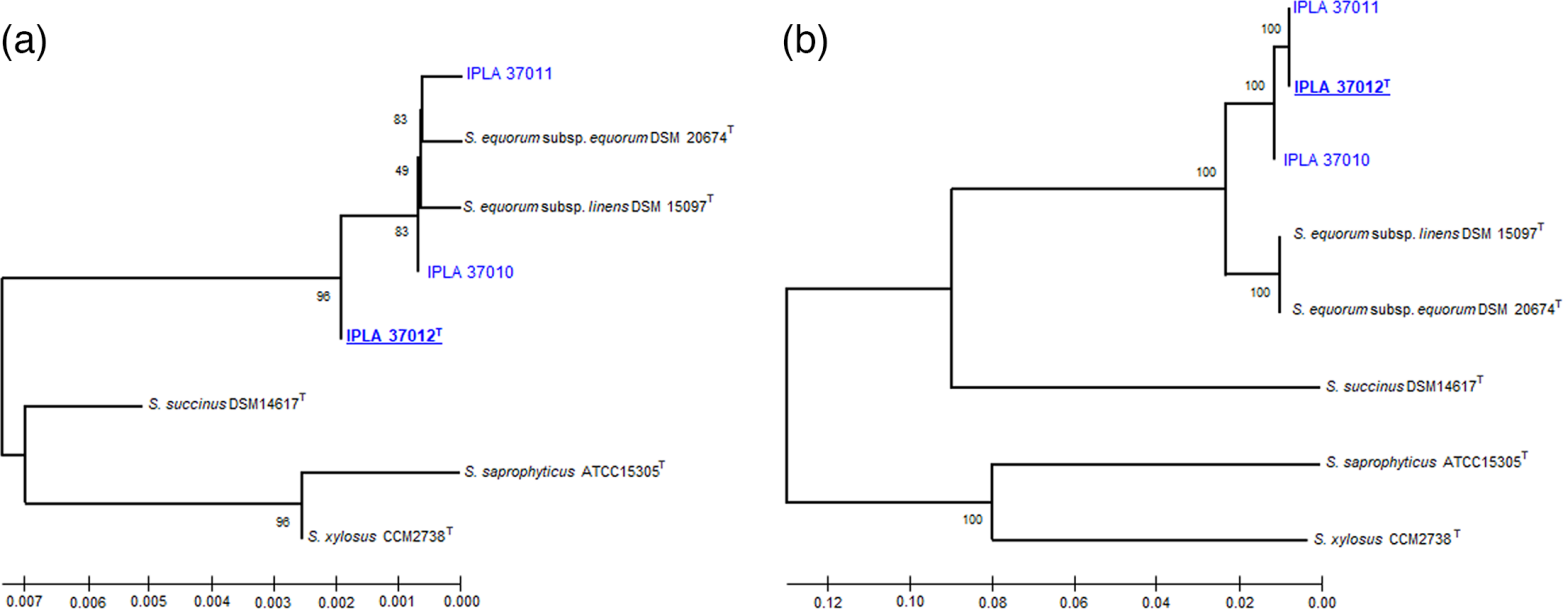
Phylogenetic trees based on gene sequences extracted from WGS data inferred by using the maximum likelihood method and the general time reversible model. The percentage of 1,000 trees in which the associated taxa clustered together is shown below the branches. Trees are drawn to scale, with branch lengths measured in the number of substitutions per site. Panel A, tree based on the complete 16S rRNA gene sequences (1,512 nt). Panel B, tree based on MLSA of concatenated nucleotide sequences from 20 loci: *aroE*, *atpA*, *ddlA*, *dnaE*, *dnaJ*, *glpF*, *gmk*, *groEL*, *mutS*, *gapA*, *gapN*, *pyrE*, *pheS*, *pta*, *recN*, *rpoA*, *rpoB*, *sodA*, *thrS* and *tuf* genes (27,765 nt).

The consensus 16S rRNA gene sequences obtained from the PCR amplicons resulted in 1,478, 1,474 and 1,465 nucleotide-long sequences for IPLA 37010, IPLA 37011 and IPLA 37012^T^, respectively. The nearly complete DNA sequences of 16S rRNA genes of the cheese strains obtained by PCR and Sanger sequencing were deposited in the NCBI database under the respective GenBank accession numbers PV547698.1, PV547699.1 and PV547700.1. The sequences were then aligned with those extracted from WGS data using the Vector NTI Align Tool. Except for a few mismatches due to heterogeneity of the ribosomal operons, there was agreement at all positions between the genomic data and amplicon-based sequences (Fig. S2). Phylogenetic analysis of the complete 16S rRNA genes of three examined strains showed their sequences to cluster with those of the type strains *S. equorum* subsp. *equorum* DSM 20674^T^ and *S. equorum* subsp. *linens* DSM 15097^T^ and to be separated from other staphylococcal species (Fig. 3a).

Given the limited usefulness of the 16S rRNA gene for identifying most staphylococcal species, phylogenetic trees were constructed for individual and concatenated sequences of 20 housekeeping genes with phylogenetic interest: *aroE*, *atpA*, *ddlA*, *dnaE*, *dnaJ*, *glpF*, *gmk*, *groEL*, *mutS*, *gapA*, *gapN*, *pyrE*, *pheS*, *pta*, *recN*, *rpoA*, *rpoB*, *sodA*, *thrS* and *tuf*. Phylogenetic trees of each of the 20 individual housekeeping genes showed the three cheese strains to form a phylogenetic cluster well separated from that of the two *S. equorum* type strains (Fig. S3). Each individual gene was associated with more genetic diversity and showed a faster evolutionary rate than the 16S rRNA gene, as indicated by the evolutionary distance scale bars. As single-gene phylogenetic trees typically have low statistical support [22,43, 44], a multilocus sequence analysis (MLSA) was performed. For this, all 20 single-gene sequences were concatenated into a ‘supergene’ containing 27,765 nucleotides in total. The phylogenetic tree derived from the concatenated sequences showed the study strains to cluster separately from the two *S. equorum* type strains (Fig. 3b). This tree, however, showed identical topology to each of the phylogenetic trees for the individual genes (Fig. S3), which is consistent with a genome-wide independent evolutionary divergence of *S. equorum* and *S. caseorum*.

A search for sequence homology to 11 *S*. *caseorum* housekeeping genes (*gapA*, *gapN*, *dnaJ*, *rpoB*, *tuf*, *aroE*, *glpF*, *gmk*, *groEL*, *mutS* and *pta*), which have been used to infer phylogenetic relationships between staphylococcal strains, was performed using the blast software program (https://blast.ncbi.nlm.nih.gov/Blast.cgi) on *S. equorum* genomes deposited at the NCBI genome database. This search identified three strains, OffWhite_SAM, EBP3087 and TMW 2.1763, mostly of whose corresponding genes showed 100% nucleotide identity to those of IPLA 37012^T^. Strains OffWhite_SAM (Accession number ASM174799v1), EBP3087 (Accession number EBP3087_S7) and TMW 2.1763 (Accession number ASM2953182v1) had been isolated from cheese rind, smoked salmon and roe salami, respectively. Further, the genomes of two strains from the NCBI genome database, isolated from a Korean ganjang sauce and identified as *Staphylococcus* spp., Mo2-6 and S9 (GenBank accession numbers CP150682.1 and CP150678.1, respectively; accessed in July 2025), showed ortho-ANI and dDDH values with the *S. caseorum* strains higher than those with the *S. equorum* type strains. Consequently, all these strains may actually belong to the new species proposed in this study, indicating the *S. caseorum* species is present in cheeses other than Cabrales and in other foods. Similarly, searches for the same DNA sequences in metagenome-assembled genomes (MAGs) of *S. equorum* from Cabrales cheese identified 11 out of 21 MAGs analysed (BioSample accession numbers SAMN23026855–SAMN23027467) [14] harbouring genes showing 100% identity to a majority of the query sequences from the *S. caseorum* strains.

### Morphology, physiology and biochemical characteristics

**Fig. 4.**
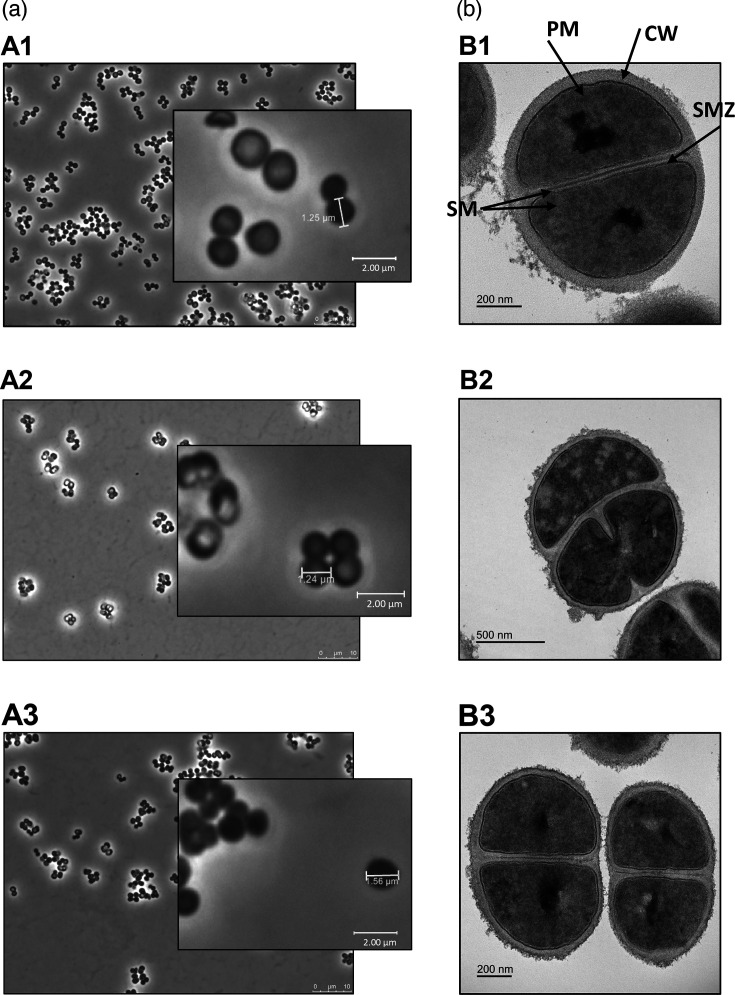
Cell morphology of strains of the newly proposed species *S. caseorum* strains IPLA 37010 (**A1, B1**), IPLA 37011 (**A2, B2**) and IPLA 37012^T^ (**A3, B3**) visualized during exponential phase (a) with an inverted optical microscope and (b) by TEM with negative strain. Cells were highly active and dividing cells were captured for all strains. Key of abbreviations: PM, plasmatic membrane; CW, cell wall; SM, septal membranes; SMZ, septal mid-zone. Scale bar represents 10 µm for the optical microscope images and 200/500 nm for those acquired by the TEM.

Bacterial cells were approximately spherical in shape as determined by the average measurements of perpendicular cell axes: 1.28±0.06×1.25±0.06 µm for IPLA 37010, 1.27±0.09×1.24±0.07 µm for IPLA 37011 and 1.40±0.14×1.41±0.1 µm for IPLA 37012^T^ (Fig. 4a). The cells formed small chains or tetrads (IPLA 37010) or small aggregates of a few cells (IPLA 37011 and IPLA 37012^T^). *S. caseorum* cells tended to be larger than those of *S. equorum* subsp. *equorum* DSM 20674^T^ (0.5–1.5 µm) [45] and *S. equorum* subsp. *linens* DSM 15097^T^ (0.9–1.4 µm) [46]. Under the electron microscope, cells of all strains appeared surrounded by a thick cell wall and were physiologically active at the time of cell harvesting (Fig. 4b). A fully completed transverse septum and incipient septa revealing ensuing division were also observed.

Strains IPLA 37010, IPLA 37011 and IPLA 37012^T^ were all catalase positive and negative for coagulase and oxidase activity. In contrast to *S. aureus* subsp. *aureus* RN4220, no nuclease activity was revealed for any of the cheese strains in toluidine-containing DNase agar plates with intact cells or heat-inactivated cell-free extracts. Nonetheless, a gene encoding a nuclease showing certain homology (61–66% amino acid identity) to *S. aureus* NucA was identified in the genome of all cheese strains. Unlike *S. aureus* and in agreement with a vast majority of CoNS species [47], all three cheese strains yielded negative results in the agglutination test for clumping factor. Strains IPLA 37010 and IPLA 37011 showed weak *α*-haemolytic activity, whereas all three examined strains were negative for *β*- and *δ*-haemolysis. All three isolates fermented mannitol, but as determined by Stoneʼs reaction for pathogenic staphylococci [48], none was capable of hydrolysing gelatin. Although reduced compared with aerated conditions, anaerobic growth was observed on BHI for all three strains, confirming the species as facultatively anaerobic, in agreement with the facultative anaerobic nature reported for most staphylococcal species [48].

At different rates, all strains grew at 5 °C (except for IPLA 37010), up to 37 °C, with 32 °C the optimal temperature. The strains also grew from pH 7.4 down to pH 5.5, with weak growth shown only by IPLA 37010 at pH 5.25. Finally, all strains grew at 15% NaCl, and all but IPLA 37011 grew at 20%; none of the strains was able to grow at 25% NaCl. The *S. caseorum* strains returned an optimal temperature at 30–32 °C, similar to that observed for *S. equorum* subsp. *equorum* DSM 20674^T^ [45] and *S. equorum* subsp. *linens* DSM 15097^T^ [46]. However, *S. caseorum* showed a narrower temperature tolerance range than *S. equorum* subsp. *linens* strains, which can grow between 6 and 40 °C [46]. S. *equorum* subsp. *equorum* has been reported to grow slowly at 37 °C and not at all at 42 °C [45]. *S. equorum* subsp. *linens* and S. *equorum* subsp. *equorum* have been reported to grow well in the presence of 13% NaCl, but weakly at 15% [46]. Under the conditions of this study, both *S. equorum* type strains grew at 20% NaCl, as the cheese strains did.

As usual for phenotypic tests in other bacterial species, wide variation was observed for carbohydrates utilization across the different *S. caseorum* strains (Table 2). In general, the fermentation profile of the strains of this study was larger than those of the two *S. equorum* type strains. All strains reduced nitrates to nitrite, returned a positive Voges–Proskauer test (acetoin production) and showed *β*-galactosidase activity. All but IPLA 37012^T^ returned a positive urease test. Alignment of all genes within the urease gene cluster (*ureABCEFGD*) revealed a point mutation in *ureF* of IPLA 37012^T^ leading to an amino acid substitution in the protein (L131P). This was the only difference at the nucleotide and its deduced amino acid sequence between the urease gene cluster of IPLA 37012^T^ and IPLA 37011, which indicates that the urease-negative phenotype of IPLA 37012^T^ is a strain-specific trait.

**Table 2.** Biochemical characteristics of *S. caseorum* strains and the type strains *S. equorum* subsp. *equorum* DSM 20674^T^ and *S. equorum* subsp. *linens* DSM 15097^T^

Strain	*S. caseorum*			*S. equorum*	
	IPLA 37010	IPLA 37011	IPLA 37012**^T^**	DSM 20674**^T^**	DSM 15097**^T^**
Carbohydrate utilization*					
*N*-Acetyl-glucosamine	++	+	+	+	−
d-Arabinose	−	+	+	+	+
l-Arabinose	+	+	+	+	+
Arbutin	+	+	−	+	−
Erythritol	−	+	+	−	−
d-Fructose	+	+	+	+	+
d-Galactose	+	+	+	+	+
Gentibiose	+	+	−	−	−
Gluconate	(+)	+	+	(+)	(+)
d-Glucose	+	+	+	+	+
Glycerol	+	+	+	+	+
d-Lactose	+	++	++	+	−
d-Mannose	++	+	+	+	+
d-Maltose	++	++	+	+	−
d-Mannitol	++	+	+	+	−
d-Ribose	−	++	++	+	−
Salicin	+	−	−	+	−
Sorbitol	−	−	−	+	+
d-Sucrose	++	+	+	+	−
d-Trehalose	++	++	+	+	−
d-Turanose	−	−	−	+	−
d-Xylose	+	+	−	+	+
**Enzyme activity**†					
*N*-Acetyl-*β*-glucosaminidase	−	−	−	5‡	−
Acid phosphatase	−	−	−	5	−
Alkaline phosphatase	5	10	5	5	5
Catalase	−	−	−	−	−
Cystine arylamidase	5	10	5	5	5
DNase	−	−	−	−	−
Esterase (C4)	>40	>40	>40	>40	>40
Esterase lipase (C8)	30	30	30	30	30
*α*-Fucosidase	−	−	5	−	−
*β*-Galactosidase	20	10	5	5	5
*β*-Glucosidase	>40	−	−	>40	−
Lipase (C14)	−	5	5	−	−
Leucine arylamidase	10	10	10	10	10
Naphtol-AS-BI-phosphoshydrolase	5	10	5	5	10
Pyrrolidonyl arylamidase	−	+	+	−	−
Urease	++	++	−	++	++
Valine arylamidase	10	10	10	10	10
**Other tests**					
Acetoin production	+	+	+	−	+
Clumping factor	−	−	−	−	−
Aesculin hydrolysis	+	+	+	+	+
Reduction of nitrate	+	++	+	++	++
Novobiocin resistance§	+ (16)	+ (8)	+ (8)	+ (32)	+ (32)

*Carbohydrate utilization includes only substrates that yield positive results with either API Staph ID 32 or API 50 CH systems.

†Enzyme activity includes only activities that yield positive results with either API Staph ID 32 or API ZYM systems.

‡Enzyme activity with the API ZYM system is given in nmol of hydrolysed substrate, ranging from 0 (−) to >40 nmol.

§Resistance with API Staph ID 32; in brackets, MIC (in µg ml−1) by broth microdilution.

Key of symbols: +, utilization or activity; (+), weak utilization; −, no utilization or activity.

The MIC of 16 antibiotics to the cheese strains has already been reported in a previous study [16]. With respect to novobiocin resistance/susceptibility – a phenotypic trait proposed for distinguishing *S. equorum* subsp. *equorum* (resistant) [45] from *S. equorum* subsp. *linens* (susceptible) [46], all strains, including the cheese isolates DSM 20674^T^ and DSM 15097^T^, proved resistant. This result, which contradicts the literature, prompted an assessment of the actual MIC of novobiocin to all five strains. An MIC value of 32 µg ml^−1^ was obtained for the type strains of the two *S. equorum* subspecies, while this value was one (for IPLA 37010) or two (for IPLA 37011 and IPLA 37012^T^) dilutions lower for the *S. caseorum* strains (Table 2). As already proposed by some authors for *S. equorum* subsp. *equorum* and *S. equorum* subsp. *linens* [20], these results suggest that the novobiocin resistance test is not suitable for distinguishing *S. equorum* from *S. caseorum*.

The results of the API Staph ID 32 were complemented with those returned by the API 50 CH and API ZYM systems. In agreement with the API Staph ID 32 results, all strains utilized d-galactose, d-glucose, d-fructose, d-mannose, d-mannitol, d-maltose, d-lactose and d-sucrose (Table 2). The strains were also positive for aesculin hydrolysis and glycerol utilization, indicating these metabolic features to be well conserved across *Staphylococcus* species [3,48]. The utilization of arbutin, erythritol, gentiobiose, salicin and d-xylose varied across the strains. In contrast to the type strains of *S. equorum* subsp. *equorum* and *S. equorum* subsp. *linens*, sorbitol was not utilized. The API ZYM system revealed a complex profile with strong, moderate and low enzyme activities (Table 2). All three strains showed strong and moderate esterase C4 and esterase lipase (C8) activity, respectively, while leucine, valine and cystine arylamidase activity was consistently weak. *β*-galactosidase and *β*-glucosidase activity varied widely across the *S. caseorum* strains, with IPLA 37010 showing the strongest activity for these enzymes. Among the activities revealed by the API ZYM system, esterase and esterase lipase activities are likely to be important for aroma and flavour formation in cheese [49].

None of the study strains produced histamine, tyramine or putrescine from any of the three amino acid precursors assayed. This agrees with the absence in their genomes of genes encoding amino acid-specific decarboxylases.

The strains showed no appreciable growth in unsupplemented milk (data not shown). Under the test conditions, the metabolic activity of the *S. caseorum* strains in tryptone-supplemented milk was very limited. With slight differences between them, all three exhibited a similar pattern of production/consumption of sugars and organic acids (Table 3). They all utilized most of the free glucose and released some galactose. In addition, all three strains produced small amounts of acetic, lactic and pyruvic acid. The amounts of citric, orotic, oxalic and uric acids before and after incubation remained the same (Table 3). In addition to providing information on possible contributions to dairy product ripening, the production/consumption of sugars and organic acids in milk may have a phylogenetic value.

**Table 3.** Sugar and organic acid profiles after growth of *S. caseorum* sp. nov. strains in UHT-treated semi-skim milk at 32 °C during 48 h

Strain	Sugar†		Organic acid*							
	Galactose	Glucose	Acetic acid‡	Citric acid	Hippuric acid	Lactic acid	Orotic acid	Oxalic acid	Pyruvic acid	Uric acid
**IPLA 37010**	12.02±2.4	3.00±0.6	11.63±0.8	173.98±1.0	0.16±0.04	26.18±3.1	8.27±0.2	0.63±0.05	11.01±1.9	1.71±0.06
**IPLA 37011**	9.87±2.1	3.10±0.3	11.83±5.3	168.84±6.2	0.98±0.2	24.92±8.3	8.59±0.6	0.66±0.03	19.53±4.2	1.54±0.01
**IPLA 37012^T^**	7.84±0.6	2.47±0.3	3.53±5.0	153.76±14.5	0.46±0.2	15.34±2.8	7.85±0.1	0.58±0.03	3.53±2.8	1.45±0.03
**Milk (control**)	7.10±1.6	7.15±2.0	nd	144.55±45.3	1.97±0.5	nd	6.84±1.7	0.65±0.06	1.63±0.5	1.45±0.4

*Results expressed as mg per 100 ml of milk.

†Lactose was not quantified because the concentration was above the calibration range; glycerol, formic acid and ethanol were not found in any of the samples.

‡Concentration of acetic acid was theoretically calculated due to the coelution with uric acid.

nd, not detected.

### Chemotaxonomic analyses

MALDI-TOF MS is a powerful tool for describing new taxa [50] and has been shown to provide a reliable and sensitive means of identifying some *Staphylococcus* species [51]. The MALDI-TOF spectra of the three strains showed no significant similarity (score<1.999) to any reference entry for a *Staphylococcus* species in the DSMZ database, including the strains of the two *S. equorum* subspecies. IPLA 37010 and IPLA 37011 returned a MALDI-TOF score of 2.34, assigning them to the same species rank. The results further showed *S. equorum* to be their closest relative (maximum scores of 1.85 and 1.98 for IPLA 37010 and IPLA 37011 with respect to *S. equorum* DSM 20675). The best score for IPLA 37012^T^ was 1.6 with IPLA 37010 – far below the ‘probable genus identification’ value of >1.7 and only 1.46, the best match with a species with a validly published name in the database, with *S. aureus* subsp. *aureus* DSM 346. Furthermore, cluster analysis of the MALDI-TOF MS spectra of the three *S. caseorum* strains did not group the strains with reference strains from the DSMZ database (Fig. S4). These results indicate a lack of *S. caseorum* species-specific spectra in the DSMZ MALDI-TOF database.

The cell-wall peptidoglycans of the cheese strains contained muramic acid and the amino acids lysine (Lys), alanine (Ala), glycine (Gly) and glutamic acid (Glu). Amino acid quantification resulted in the following molar ratios: IPLA 37010, 1.4 Ala:2.9 Gly:1.0 Glu:1.7 Lys; IPLA 37011, 1.8 Ala:3.3 Gly:1.0 Glu:1.6 Lys; and IPLA 37012^T^, 1.9 Ala:3.4 Gly:1.0 Glu:0.8 Lys. The identity of all four amino acids was confirmed by agreement with the gas-chromatographic retention times of standards and by the characteristic mass-spectrometric fragment ions of their derivatives. Analysis of the enantiomers of the hydrolysates revealed l-Lys and d-Glu in all three samples. After hydrolysis under milder conditions (100 °C, 4 N HCl, 45 min), the peptides detected (identical in the three strains) were as follows: Ala-Glu, Lys-Ala, Lys-Gly, Ala-Lys-Gly, Ala-Ala-Lys-Gly, Ala-Glu-Lys-Ala/-Gly, Lys-Ala-Ala, muramic acid-Ala-Glu, Lys-Gly-Gly, Glu-Lys-Gly_3_. In accordance with the DSMZ database (https://www.peptidoglycan-types.info/), it was concluded that the three strains have the peptidoglycan type A3*α* l-Lys–l-Lys–Gly_3-4_; variant A11.2. The number of glycine residues in the interpeptide chain can vary depending on culture conditions, and no complete interpeptide bridge was detected. By way of comparison, the peptidoglycan type of *S. equorum* subsp. *equorum* DSM 20674^T^ is Lys-Gly_5-6_ [45] and that of *S. equorum* subsp. *linens* DSM 15097^T^ is l-Lys-Gly_5_ [46].

In conclusion, MALDI-TOF and peptidoglycan analysis further reinforce the idea that the strains of this study form a separate species taxon from *S. equorum*.

## Description of *Staphylococcus caseorum* sp. nov.

*Staphylococcus caseorum* (ca.se.o’rum. L. gen. pl. n. *caseorum*, of cheeses). Cells are Gram-positive, facultatively anaerobic, non-motile, spherical (average diameter 1.31±0.1 µm), occurring singly, in pairs or in short chains. After 48 h at 32 °C on BHI agar plates, colonies are regular, whitish, opaque, semi-glossy and 2–3 mm in diameter. Strains showed weak *α*-haemolytic activity, positive reaction in the catalase test and negative for coagulase, oxidase and nuclease activity. Further, a negative agglutination test for clumping factor was also obtained. Growth was obtained aerobically between 5 and 37 °C, at pH 5.5 and in the presence of 20% NaCl. Optimal growth occurred at 32 °C at an NaCl concentration of 2.5–5%. In API 20 Staph ID 32 and API ZYM, nitrate is reduced, aesculin hydrolysed and acetoin is produced. A positive enzymatic reaction was observed for *β*-galactosidase, arginine arylamidase, arginine dihydrolase, *β*-glucuronidase and ornithine decarboxylase. Urease activity is considered positive in the species, although negative strains due to specific mutations in urease genes (as seen in IPLA 37012^T^) may occur. In API 20 Staph ID 32 and API 50 CH, acid was formed from l-arabinose, d-galactose, *N*-acetyl-glucosamine, d-glucose, gluconate, glycerol, d-fructose, d-lactose, d-mannose, d-maltose, d-mannitol, d-sucrose and d-trehalose. It does not form acid from d-adonitol, amygdalin, d- and l-arabitol, d-cellobiose, dulcitol, inositol, inulin, 2-ketogluconate, 5-ketogluconate, d- and l-fucose, glycogen, methyl-*α*-d-glucopyranoside, d-lyxose, methyl-*α*-d-mannopyranoside, d-melibiose, d-melezitose, d-raffinose, l-rhamnose, sorbitol, l-sorbose, starch, d-tagatose, d-turanose, methyl-*β*-d-xylopyranoside, xylitol or l-xylose, while strain-to-strain variation was shown for the utilization of d-arabinose, arbutin, gentibiose, d-ribose, salicin and d-xylose. Resistance to novobiocin was observed. The biogenic amines histamine, tyramine and putrescine were not produced; dedicated amino acid-specific decarboxylases in the genome were not found. The peptidoglycan was of type A3*α* l-Lys–l-Lys–Gly_3-4_; variant A11.2. Strain IPLA 37012^T^ is the type strain (=DSM 117647^T^, =CECT 31035^T^). The nearly complete 16S rRNA gene sequence of IPLA37012^T^ was deposited in GenBank under accession number PV547700.1. The long-read WGS data of IPLA37012^T^ and its hybrid Illumina and PacBio assembled genome were deposited in the NCBI Sequence Read Archive database (https://www.ncbi.nlm.nih.gov/sra) under BioProject PRJNA1253107 (SRR33234683-85) and Genome Assembly ASM4994319v1, respectively.

## Supplementary material

10.1099/ijsem.0.007144 Uncited Supplementary Material 1.

## Abbreviations

BHI: brain heart infusion
BV-BRC: Bacterial and Viral Bioinformatics Resource Center
CDSs: coding sequences
CoNS: coagulase-negative
CoPS: coagulase-positive
CRISPR-Cas: clustered regularly interspaced short palindromic repeats-CRISPR associated
dDDH: digital DNA–DNA hybridization
GBDP: genome blast distance phylogeny
MAGs: metagenome-assembled genomes
MALDI-TOF: matrix-assisted laser desorption/ionization time-of-flight
MLSA: multilocus sequence analysis
NCBI: National Center for Biotechnology Information
ortho-ANI: ortho-average nucleotide identity
PDO: protected designation of origin
RAST: rapid annotations using subsystems technology
RASTtk: RAST tool kit
TEM: transmission electron microscope
TYGS: type (strain) genome server
UHT: ultrahigh temperature
VFDB: virulence factor database
WGS: whole-genome sequencing
