# Supplementary material for: Staphylococcus caseorum sp. nov., a new species isolated from Spanish traditional, blue-veined Cabrales cheese
Source: Int J Syst Evol Microbiol. 2026 Apr 27;76(4):007144. doi: 10.1099/ijsem.0.007144 (PMC13128282; doi:10.1099/ijsem.0.007144)
Supplement: Uncited Supplementary Material 1. [file ijsem-76-07144-s001.pdf]

Staphylococcus caseorum sp. nov., a new species isolated from Spanish traditional, blue-veined Cabrales cheese

Lucía Vázquez[1,2], Javier Rodríguez[1,2], Ana Belén Flórez[1,2], and Baltasar Mayo[1,2]

[1]Departamento de Microbiología y Bioquímica, Instituto de Productos Lácteos de Asturias (IPLA), Consejo Superior de Investigaciones Científicas (CSIC), Francisco Pintado Fe, 26, 33011-Oviedo, Spain

[2]Instituto de Investigación Sanitaria del Principado de Asturias (ISPA), Avenida de Roma s/n, 33011-Oviedo, Spain

Running title: *Staphylococcus caseorum* sp. nov., from Cabrales cheese

Keywords: *Staphylococcus*, coagulase-negative staphylococci, cheese, blue-veined cheese, cheese microbiota, starters, Cabrales

International Journal of Systematic and Evolutionary Microbiology

Supplementary Material

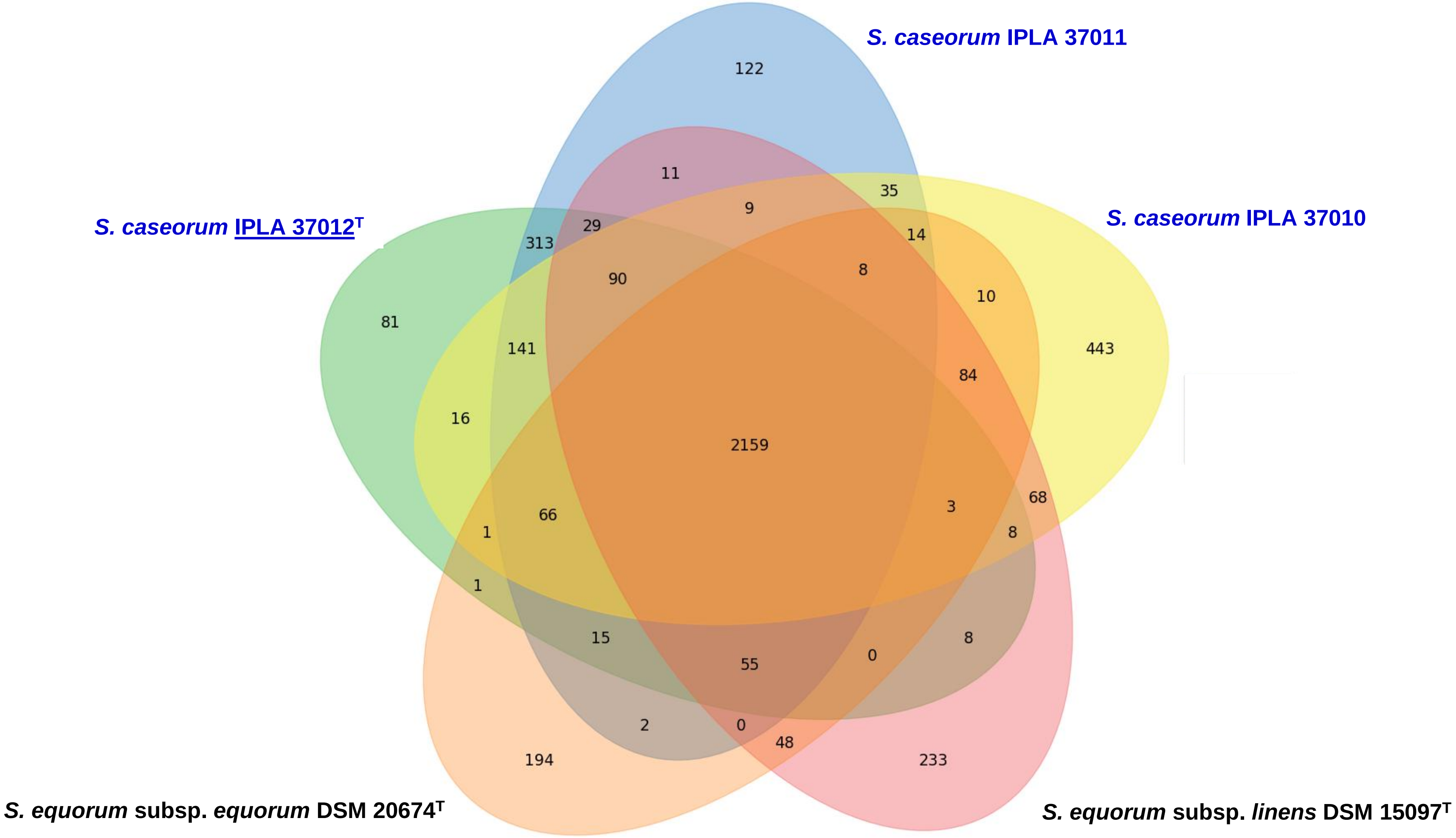

Supplementary figure 1.- Venn diagram showing the pan-genome of three strains of *Staphylococcus caseorum* described in this study and the type strains of *Staphylococcus equorum* subsp. *equorum* DSM 20674 **T** and *S. equorum* subsp. *linens* DSM 15097 T. Overlapping regions represent common CDSs shared between the different genomes. Numbers inside the regions indicate CDSs shared between genomes or strain-specific CDSs. Colour key: in yellow, *S. caseorum* IPLA 37010; in blue, *S. caseorum* IPLA 37011; in green. *S. caseorum* IPLA 37012T; in pink, *S. equorum* subsp. *linens* DSM 15097 T; in orange, *S. equorum* subsp. *equorum* DSM 20674 **T**. Comparative genome analysis between cheese strains and S. equorum type strains was carried out by using an inhouse-developed Phyton-based algorithm and visualized with Matplotlib (https://matplotlib.org/).

Supplementary figure 2.- Sequences of the16S rRNA gene from the *S. caseorum* strains of this study obtained by alignment of sequences of the PCR amplicons using primers 27F, 1492R, Y1, Y2, and 968F. Sequences were assembled and then aligned with those extracted from WGS data. In blue, green and pink sequences of the primers 27F-1492R, 27F-1492R and 968F. In yellow, differences in sequence. Nucleotides in front of sequences from amplicons are also highlighted.

Strain IPLA 37010 (1,478 nucleotides):

Amplicon-derived consensus sequence:

```
GCTGGCGGCGTGCCTAATACATGCAAGTCGAGCGAACGGATAAGGAGCTTGCTCCTTTGAAGTTAGCGGCGGACGGGTGAG
TAACACGTGGGTAACCTACCTATAAGACTGGAATAACTTCGGGAAACCGGAGCTAATGCCGGATAACATTTGGAACCGCAT
GGTTCTAAAGTAAAAGATGGTTTTGCTATCACTTATAGATGGACCCGCGCCGTATTAGCTAGTTGGTAAGGTAACGGCTTA
CCAAGGCAACGATACGTAGCCGACCTGAGAGGGTGATCGGCCACACTGGAACTGAGACACGGTCCAGACTCCTACGGGAGG
CAGCAGTAGGGAATCTTCCGCAATGGACGAAAGTCTGACGGAGCAACGCCGCGTGAGTGATGAAGGTTTTCGGATCGTAAA
ACTCTGTTATTAGGGAAGAACAAATGTGTAAGTAACTGTGCACATCTTGACGGTACCTAATCAGAAAGCCACGGCTAACTA
CGTGCCAGCAGCCGCGGTAATACGTAGGTGGCAAGCGTTATCCGGAATTATTGGGCGTAAAGCGCGCGTAGGCGGTTTCTT
AAGTCTGATGTGAAAGCCCACGGCTCAACCGTGGAGGGTCATTGGAAACTGGGAAACTTGAGTACAGAAGAGGAAAGTGGA
ATTCCATGTGTAGCGGTGAAATGCGCAGAGATATGGAGGAACACCAGTGGCGAAGGCGACTTTCTGGTCTGTAACTGACGC
TGATGTGCGAAAGCGTGGGGATCAAACAGGATTAGATACCCTGGTAGTCCACGCCGTAAACGATGAGTGCTAAGTGTTAGG
GGGTTTCCGCCCCTTAGTGCTGCAGCTAACGCATTAAGCACTCCGCCTGGGGAGTACGACCGCAAGGTTGAAACTCAAAGG
AATTGACGGGGACCCGCACAAGCGGTGGAGCATGTGGTTTAATTCGAAGCAACGCGAAGAACCTTACCAAATCTTGACATC
CTTTGAAAACTCTAGAGATAGAGCCTTCCCCTTCGGGGGACAAAGTGACAGGTGGTGCATGGTTGTCGTCAGCTCGTGTCG
TGAGATGTTGGGTTAAGTCCCGCAACGAGCGCAACCCTTAAACTTAGTTGCCAGCATTTAGTTGGGCACTCTAGGTTGACT
GCCGGTGACAAACCGGAGGAAGGTGGGGATGACGTCAAATCATCATGCCCCTTATGATTTGGGCTACACACGTGCTACAAT
GGACAATACAAAGGGCAGCTAAACCGCGAGGTCATGCAAATCCCATAAAGTTGTTCTCAGTTCGGATTGTAGTCTGCTAAC
TCGACTACATGAAGCTGGAATCGCTAGTAATCGTAGATCAGCATGCTACGGTGTAATACGTTCCCGGGTCTTGTACACACC
GCCCGTCACACCACGAGAGTTTGTAACACCCGAAGCCGGTGGAGTAACCATTTATGGAGCTAGCCGTCGAAGGTGGGACAA
ATGATTGGGGGTGAAGTCGG
```

Alignment amplicon-derived sequence vs WGS data:

```
                              1                                                50
IPLA37010_Consensus      (1)  --------------------------------------------GCTGGC
IPLA37010_16Sgenome      (1)  TCATAATTCATTTATGGAGAGTTTGATCCTGGCTCAGGATGAACGCTGGC
                              51                                              100
IPLA37010_Consensus      (7)  GGCGTGCCTAATACATGCAAGTCGAGCGAACGGATAAGGAGCTTGCTCCT
IPLA37010_16Sgenome     (51)  GGCGTGCCTAATACATGCAAGTCGAGCGAACGGATAAGGAGCTTGCTCCT
                              101                                             150
IPLA37010_Consensus     (57)  TTGAAGTTAGCGGCGGACGGGTGAGTAACACGTGGGTAACCTACCTATAA
IPLA37010_16Sgenome    (101)  TTGAAGTTAGCGGCGGACGGGTGAGTAACACGTGGGTAACCTACCTATAA
                              151                                             200
IPLA37010_Consensus    (107)  GACTGGAATAACTTCGGGAAACCGGAGCTAATGCCGGATAACATTTGGAA
IPLA37010_16Sgenome    (151)  GACTGGAATAACTTCGGGAAACCGGAGCTAATGCCGGATAACATTTGGAA
                              201                                             250
IPLA37010_Consensus    (157)  CCGCATGGTTCTAAAGTAAAAGATGGTTTTGCTATCACTTATAGATGGAC
IPLA37010_16Sgenome    (201)  CCGCATGGTTCTAAAGTAAAAGATGGTTTTGCTATCACTTATAGATGGAC
                              251                                             300
IPLA37010_Consensus    (207)  CCGCGCCGTATTAGCTAGTTGGTAAGGTAACGGCTTACCAAGGCAACGAT
IPLA37010_16Sgenome    (251)  CCGCGCCGTATTAGCTAGTTGGTAAGGTAACGGCTTACCAAGGCAACGAT
                              301                                             350
IPLA37010_Consensus    (257)  ACGTAGCCGACCTGAGAGGGTGATCGGCCACACTGGAACTGAGACACGGT
IPLA37010_16Sgenome    (301)  ACGTAGCCGACCTGAGAGGGTGATCGGCCACACTGGAACTGAGACACGGT
                              351                                             400
IPLA37010_Consensus    (307)  CCAGACTCCTACGGGAGGCAGCAGTAGGGAATCTTCCGCAATGGACGAAA
IPLA37010_16Sgenome    (351)  CCAGACTCCTACGGGAGGCAGCAGTAGGGAATCTTCCGCAATGGACGAAA
                              401                                             450
IPLA37010_Consensus    (357)  GTCTGACGGAGCAACGCCGCGTGAGTGATGAAGGTTTTCGGATCGTAAAA
```

```
IPLA37010_16Sgenome   (401) GTCTGACGGAGCAACGCCGCGTGAGTGATGAAGGTTTTCGGATCGTAAAA
                            451                                              500
IPLA37010_Consensus   (407) CTCTGTTATTAGGGAAGAACAAATGTGTAAGTAACTGTGCACATCTTGAC
IPLA37010_16Sgenome   (451) CTCTGTTATTAGGGAAGAACAAATGTGTAAGTAACTGTGCACATCTTGAC
                            501                                              550
IPLA37010_Consensus   (457) GGTACCTAATCAGAAAGCCACGGCTAACTACGTGCCAGCAGCCGCGGTAA
IPLA37010_16Sgenome   (501) GGTACCTAATCAGAAAGCCACGGCTAACTACGTGCCAGCAGCCGCGGTAA
                            551                                              600
IPLA37010_Consensus   (507) TACGTAGGTGGCAAGCGTTATCCGGAATTATTGGGCGTAAAGCGCGCGTA
IPLA37010_16Sgenome   (551) TACGTAGGTGGCAAGCGTTATCCGGAATTATTGGGCGTAAAGCGCGCGTA
                            601                                              650
IPLA37010_Consensus   (557) GGCGGTTTCTTAAGTCTGATGTGAAAGCCCACGGCTCAACCGTGGAGGGT
IPLA37010_16Sgenome   (601) GGCGGTTTCTTAAGTCTGATGTGAAAGCCCACGGCTCAACCGTGGAGGGT
                            651                                              700
IPLA37010_Consensus   (607) CATTGGAAACTGGGAAACTTGAGTACAGAAGAGGAAAGTGGAATTCCATG
IPLA37010_16Sgenome   (651) CATTGGAAACTGGGAAACTTGAGTACAGAAGAGGAAAGTGGAATTCCATG
                            701                                              750
IPLA37010_Consensus   (657) TGTAGCGGTGAAATGCGCAGAGATATGGAGGAACACCAGTGGCGAAGGCG
IPLA37010_16Sgenome   (701) TGTAGCGGTGAAATGCGCAGAGATATGGAGGAACACCAGTGGCGAAGGCG
                            751                                              800
IPLA37010_Consensus   (707) ACTTTCTGGTCTGTAACTGACGCTGATGTGCGAAAGCGTGGGGATCAAAC
IPLA37010_16Sgenome   (751) ACTTTCTGGTCTGTAACTGACGCTGATGTGCGAAAGCGTGGGGATCAAAC
                            801                                              850
IPLA37010_Consensus   (757) AGGATTAGATACCCTGGTAGTCCACGCCGTAAACGATGAGTGCTAAGTGT
IPLA37010_16Sgenome   (801) AGGATTAGATACCCTGGTAGTCCACGCCGTAAACGATGAGTGCTAAGTGT
                            851                                              900
IPLA37010_Consensus   (807) TAGGGGGTTTCCGCCCCTTAGTGCTGCAGCTAACGCATTAAGCACTCCGC
IPLA37010_16Sgenome   (851) TAGGGGGTTTCCGCCCCTTAGTGCTGCAGCTAACGCATTAAGCACTCCGC
                            901                                              950
IPLA37010_Consensus   (857) CTGGGGAGTACGACCGCAAGGTTGAAACTCAAAGGAATTGACGGGGACCC
IPLA37010_16Sgenome   (901) CTGGGGAGTACGACCGCAAGGTTGAAACTCAAAGGAATTGACGGGGACCC
                            951                                             1000
IPLA37010_Consensus   (907) GCACAAGCGGTGGAGCATGTGGTTTAATTCGAAGCAACGCGAAGAACCTT
IPLA37010_16Sgenome   (951) GCACAAGCGGTGGAGCATGTGGTTTAATTCGAAGCAACGCGAAGAACCTT
                            1001                                            1050
IPLA37010_Consensus   (957) ACCAAATCTTGACATCCTTTGAAAACTCTAGAGATAGAGCCTTCCCCTTC
IPLA37010_16Sgenome  (1001) ACCAAATCTTGACATCCTTTGAAAACTCTAGAGATAGAGCCTTCCCCTTC
                            1051                                            1100
IPLA37010_Consensus  (1007) GGGGGACAAAGTGACAGGTGGTGCATGGTTGTCGTCAGCTCGTGTCGTGA
IPLA37010_16Sgenome  (1051) GGGGGACAAAGTGACAGGTGGTGCATGGTTGTCGTCAGCTCGTGTCGTGA
                            1101                                            1150
IPLA37010_Consensus  (1057) GATGTTGGGTTAAGTCCCGCAACGAGCGCAACCCTTAAACTTAGTTGCCA
IPLA37010_16Sgenome  (1101) GATGTTGGGTTAAGTCCCGCAACGAGCGCAACCCTTAAACTTAGTTGCCA
                            1151                                            1200
IPLA37010_Consensus  (1107) GCATTTAGTTGGGCACTCTAGGTTGACTGCCGGTGACAAACCGGAGGAAG
IPLA37010_16Sgenome  (1151) GCATTTAGTTGGGCACTCTAGGTTGACTGCCGGTGACAAACCGGAGGAAG
                            1201                                            1250
IPLA37010_Consensus  (1157) GTGGGGATGACGTCAAATCATCATGCCCCTTATGATTTGGGCTACACACG
IPLA37010_16Sgenome  (1201) GTGGGGATGACGTCAAATCATCATGCCCCTTATGATTTGGGCTACACACG
                            1251                                            1300
IPLA37010_Consensus  (1207) TGCTACAATGGACAATACAAAGGGCAGCTAAACCGCGAGGTCATGCAAAT
IPLA37010_16Sgenome  (1251) TGCTACAATGGACAATACAAAGGGCAGCTAAACCGCGAGGTCATGCAAAT
                            1301                                            1350
IPLA37010_Consensus  (1257) CCCATAAAGTTGTTCTCAGTTCGGATTGTAGTCTGCTAACTCGACTACAT
IPLA37010_16Sgenome  (1301) CCCATAAAGTTGTTCTCAGTTCGGATTGTAGTCTGC-AACTCGACTACAT
                            1351                                            1400
IPLA37010_Consensus  (1307) GAAGCTGGAATCGCTAGTAATCGTAGATCAGCATGCTACGGTGTAATACG
IPLA37010_16Sgenome  (1350) GAAGCTGGAATCGCTAGTAATCGTAGATCAGCATGCTACGGTG-AATACG
                            1401                                            1450
IPLA37010_Consensus  (1357) TTCCCGGGTCTTGTACACACCGCCCGTCACACCACGAGAGTTTGTAACAC
IPLA37010_16Sgenome  (1399) TTCCCGGGTCTTGTACACACCGCCCGTCACACCACGAGAGTTTGTAACAC
                            1451                                            1500
IPLA37010_Consensus  (1407) CCGAAGCCGGTGGAGTAACCATTTATGGAGCTAGCCGTCGAAGGTGGGAC
```

```
IPLA37010_16Sgenome   (1449) CCGAAGCCGGTGGAGTAACCATTTATGGAGCTAGCCGTCGAAGGTGGGAC
                             1501                                           1550
IPLA37010_Consensus   (1457) AAATGATTGGGGGTGAAGTCGG----------------------------
IPLA37010_16Sgenome   (1499) AAATGATTGGGG-TGAAGTCGTAACAAGGTAGCCGTATCGGAAGGTGCGG
                             1551              1569
IPLA37010_Consensus   (1479) -------------------
IPLA37010_16Sgenome   (1548) CTGGATCACCTCCTTTCTA
```

Strain IPLA 37011 (1474 nucleotides):

Amplicon-derived consensus sequence:

```
GCGGCGGCGTGCCTAATACATGCAAGTCGAGCGAACGGATAAGGAGCTTGCTCCTTTGAAGTTAGCGGCGGACGGGTGAGT
AACACGTGGGTAACCTACCTATAAGACTGGAATAACTTCGGGAAACCGGAGCTAATGCCGGATAACATTTGGAACCGCATG
GTTCTAAAGTAAAAGTATGGTTTTGCTATCACTTATAGATGGACCCGCGCCGTATTAGCTAGTTGGTAAGGTAACGGCTTA
CCAAGGCAACGATACGTAGCCGACCTGAGAGGGTGATCGGCCACACTGGAACTGAGACACGGTCCAGACTCCTACGGGAGG
CAGCAGTAGGGAATCTTCCGCAATGGACGAAAGTCTGACGGAGCAACGCCGCGTGAGTGATGAAGGTTTTCGGATCGTAAA
ACTCTGTTATTAGGGAAGAACAAATGCGTAAGTAACTGTGCGCATCTTGACGGTACCTAATCAGAAAGCCACGGCTAACTA
CGTGCCAGCAGCCGCGGTAATACGTAGGTGGCAAGCGTTATCCGGAATTATTGGGCGTAAAGCGCGCGTAGGCGGTTTCTT
AAGTCTGATGTGAAAGCCCACGGCTCAACCGTGGAGGGTCATTGGAAACTGGGAAACTTGAGTACAGAAGAGGAAAGTGGA
ATTCCATGTGTAGCGGTGAAATGCGCAGAGATATGGAGGAACACCAGTGGCGAAGGCGACTTTCTGGTCTGTAACTGACGC
TGATGTGCGAAAGCGTGGGGATCAAACAGGATTAGATACCCTGGTAGTCCACGCCGTAAACGATGAGTGCTAAGTGTTTAG
GGGGTTTCCGCCCCTTAGTGCTGCAGCTAACGCATTAAGCACTCCGCCTGGGGAGTACGACCGCAAGGTTGAAACTCAAAG
GAATTGACGGGGACCCGCACAAGCGGTGGAGCATGTGGTTTAATTCGAAGCAACGCGAAGAACCTTACCAAATCTTGACAT
CCTTTGAAAACTCTAGAGATAGAGCCTTCCCCTTCGGGGGACAAAGTGACAGGTGGTGCATGGTTGTCGTCAGCTCGTGTC
GTGAGATGTTGGGTTAAGTCCCGCAACGAGCGCAACCCTTAAACTTAGTTGCCAGCATTTAGTTGGGCACTCTAGGTTGAC
TGCCGGTGACAAACCGGAGGAAGGTGGGGATGACGTCAAATCATCATGCCCCTTATGATTTGGGCTACACACGTGCTACAA
TGGACAATACAAAGGGCAGCTAAACCGCGAGGTCATGCAAATCCCATAAAGTTGTTCTCAGTTCGGATTGTAGTCTGCAAC
TCGACTACATGAAGCTGGAATCGCTAGTAATCGTAGATCAGCATGCTACGGTGAATACGTTCCCGGGTCTTGTACACACCG
CCCGTCACACCACGAGAGTTTGTAACACCCGAAGCCGGTGGAGTAACCATTTATGGAGCTAGCCGTCGAAGGTGGGACAAA
TGATTGGGGTGAAGTC
```

Alignment amplicon-derived sequence vs WGS data:

```
                               1                                               50
IPLA37011_Consensus     (1) --------------------------------------------GC-GGC
11A1I_16Sgene_genome    (1) TCATAATTCATTTATGGAGAGTTTGATCCTGGCTCAGGATGAACGCTGGC
                              51                                             100
IPLA37011_Consensus     (6) GGCGTGCCTAATACATGCAAGTCGAGCGAACGGATAAGGAGCTTGCTCCT
11A1I_16Sgene_genome   (51) GGCGTGCCTAATACATGCAAGTCGAGCGAACGGATAAGGAGCTTGCTCCT
                              101                                            150
IPLA37011_Consensus    (56) TTGAAGTTAGCGGCGGACGGGTGAGTAACACGTGGGTAACCTACCTATAA
11A1I_16Sgene_genome  (101) TTGAAGTTAGCGGCGGACGGGTGAGTAACACGTGGGTAACCTACCTATAA
                              151                                            200
IPLA37011_Consensus   (106) GACTGGAATAACTTCGGGAAACCGGAGCTAATGCCGGATAACATTTGGAA
IPLA37011_16Sgenome   (151) GACTGGAATAACTTCGGGAAACCGGAGCTAATGCCGGATAACATTTGGAA
                              201                                            250
IPLA37011_Consensus   (156) CCGCATGGTTCTAAAGTAAAAGTATGGTTTTGCTATCACTTATAGATGGA
IPLA37011_16Sgenome   (201) CCGCATGGTTCTAAAGTAAAAG-ATGGTTTTGCTATCACTTATAGATGGA
                              251                                            300
IPLA37011_Consensus   (206) CCCGCGCCGTATTAGCTAGTTGGTAAGGTAACGGCTTACCAAGGCAACGA
IPLA37011_16Sgenome   (250) CCCGCGCCGTATTAGCTAGTTGGTAAGGTAACGGCTTACCAAGGCAACGA
                              301                                            350
IPLA37011_Consensus   (256) TACGTAGCCGACCTGAGAGGGTGATCGGCCACACTGGAACTGAGACACGG
IPLA37011_16Sgenome   (300) TACGTAGCCGACCTGAGAGGGTGATCGGCCACACTGGAACTGAGACACGG
                              351                                            400
IPLA37011_Consensus   (306) TCCAGACTCCTACGGGAGGCAGCAGTAGGGAATCTTCCGCAATGGACGAA
IPLA37011_16Sgenome   (350) TCCAGACTCCTACGGGAGGCAGCAGTAGGGAATCTTCCGCAATGGACGAA
```

```
                                  401                                               450
IPLA37011_Consensus   (356) AGTCTGACGGAGCAACGCCGCGTGAGTGATGAAGGTTTTCGGATCGTAAA
IPLA37011_16Sgenome   (400) AGTCTGACGGAGCAACGCCGCGTGAGTGATGAAGGTTTTCGGATCGTAAA
                                  451                                               500
IPLA37011_Consensus   (406) ACTCTGTTATTAGGGAAGAACAAATGCGTAAGTAACTGTGCGCATCTTGA
IPLA37011_16Sgenome   (450) ACTCTGTTATTAGGGAAGAACAAATGCGTAAGTAACTGTGCGCATCTTGA
                                  501                                               550
IPLA37011_Consensus   (456) CGGTACCTAATCAGAAAGCCACGGCTAACTACGTGCCAGCAGCCGCGGTA
IPLA37011_16Sgenome   (500) CGGTACCTAATCAGAAAGCCACGGCTAACTACGTGCCAGCAGCCGCGGTA
                                  551                                               600
IPLA37011_Consensus   (506) ATACGTAGGTGGCAAGCGTTATCCGGAATTATTGGGCGTAAAGCGCGCGT
IPLA37011_16Sgenome   (550) ATACGTAGGTGGCAAGCGTTATCCGGAATTATTGGGCGTAAAGCGCGCGT
                                  601                                               650
IPLA37011_Consensus   (556) AGGCGGTTTCTTAAGTCTGATGTGAAAGCCCACGGCTCAACCGTGGAGGG
IPLA37011_16Sgenome   (600) AGGCGGTTTCTTAAGTCTGATGTGAAAGCCCACGGCTCAACCGTGGAGGG
                                  651                                               700
IPLA37011_Consensus   (606) TCATTGGAAACTGGGAAACTTGAGTACAGAAGAGGAAAGTGGAATTCCAT
IPLA37011_16Sgenome   (650) TCATTGGAAACTGGGAAACTTGAGTACAGAAGAGGAAAGTGGAATTCCAT
                                  701                                               750
IPLA37011_Consensus   (656) GTGTAGCGGTGAAATGCGCAGAGATATGGAGGAACACCAGTGGCGAAGGC
IPLA37011_16Sgenome   (700) GTGTAGCGGTGAAATGCGCAGAGATATGGAGGAACACCAGTGGCGAAGGC
                                  751                                               800
IPLA37011_Consensus   (706) GACTTTCTGGTCTGTAACTGACGCTGATGTGCGAAAGCGTGGGGATCAAA
IPLA37011_16Sgenome   (750) GACTTTCTGGTCTGTAACTGACGCTGATGTGCGAAAGCGTGGGGATCAAA
                                  801                                               850
IPLA37011_Consensus   (756) CAGGATTAGATACCCTGGTAGTCCACGCCGTAAACGATGAGTGCTAAGTG
IPLA37011_16Sgenome   (800) CAGGATTAGATACCCTGGTAGTCCACGCCGTAAACGATGAGTGCTAAGTG
                                  851                                               900
IPLA37011_Consensus   (806) TTTAGGGGGTTTCCGCCCCTTAGTGCTGCAGCTAACGCATTAAGCACTCC
IPLA37011_16Sgenome   (850) TT-AGGGGGTTTCCGCCCCTTAGTGCTGCAGCTAACGCATTAAGCACTCC
                                  901                                               950
IPLA37011_Consensus   (856) GCCTGGGGAGTACGACCGCAAGGTTGAAACTCAAAGGAATTGACGGGGAC
IPLA37011_16Sgenome   (899) GCCTGGGGAGTACGACCGCAAGGTTGAAACTCAAAGGAATTGACGGGGAC
                                  951                                              1000
IPLA37011_Consensus   (906) CCGCACAAGCGGTGGAGCATGTGGTTTAATTCGAAGCAACGCGAAGAACC
IPLA37011_16Sgenome   (949) CCGCACAAGCGGTGGAGCATGTGGTTTAATTCGAAGCAACGCGAAGAACC
                                  1001                                             1050
IPLA37011_Consensus   (956) TTACCAAATCTTGACATCCTTTGAAAACTCTAGAGATAGAGCCTTCCCCT
IPLA37011_16Sgenome   (999) TTACCAAATCTTGACATCCTTTGAAAACTCTAGAGATAGAGCCTTCCCCT
                                  1051                                             1100
IPLA37011_Consensus  (1006) TCGGGGGACAAAGTGACAGGTGGTGCATGGTTGTCGTCAGCTCGTGTCGT
IPLA37011_16Sgenome  (1049) TCGGGGGACAAAGTGACAGGTGGTGCATGGTTGTCGTCAGCTCGTGTCGT
                                  1101                                             1150
IPLA37011_Consensus  (1056) GAGATGTTGGGTTAAGTCCCGCAACGAGCGCAACCCTTAAACTTAGTTGC
IPLA37011_16Sgenome  (1099) GAGATGTTGGGTTAAGTCCCGCAACGAGCGCAACCCTTAAACTTAGTTGC
                                  1151                                             1200
IPLA37011_Consensus  (1106) CAGCATTTAGTTGGGCACTCTAGGTTGACTGCCGGTGACAAACCGGAGGA
IPLA37011_16Sgenome  (1149) CAGCATTTAGTTGGGCACTCTAGGTTGACTGCCGGTGACAAACCGGAGGA
                                  1201                                             1250
IPLA37011_Consensus  (1156) AGGTGGGGATGACGTCAAATCATCATGCCCCTTATGATTTGGGCTACACA
IPLA37011_16Sgenome  (1199) AGGTGGGGATGACGTCAAATCATCATGCCCCTTATGATTTGGGCTACACA
                                  1251                                             1300
IPLA37011_Consensus  (1206) CGTGCTACAATGGACAATACAAAGGGCAGCTAAACCGCGAGGTCATGCAA
IPLA37011_16Sgenome  (1249) CGTGCTACAATGGACAATACAAAGGGCAGCTAAACCGCGAGGTCATGCAA
                                  1301                                             1350
IPLA37011_Consensus  (1256) ATCCCATAAAGTTGTTCTCAGTTCGGATTGTAGTCTGCAACTCGACTACA
IPLA37011_16Sgenome  (1299) ATCCCATAAAGTTGTTCTCAGTTCGGATTGTAGTCTGCAACTCGACTACA
                                  1351                                             1400
IPLA37011_Consensus  (1306) TGAAGCTGGAATCGCTAGTAATCGTAGATCAGCATGCTACGGTGAATACG
IPLA37011_16Sgenome  (1349) TGAAGCTGGAATCGCTAGTAATCGTAGATCAGCATGCTACGGTGAATACG
                                  1401                                             1450
IPLA37011_Consensus  (1356) TTCCCGGGTCTTGTACACACCGCCCGTCACACCACGAGAGTTTGTAACAC
IPLA37011_16Sgenome  (1399) TTCCCGGGTCTTGTACACACCGCCCGTCACACCACGAGAGTTTGTAACAC
```

```
                                  1451                                           1500
IPLA37011_Consensus     (1406)  CCGAAGCCGGTGGAGTAACCATTTATGGAGCTAGCCGTCGAAGGTGGGAC
IPLA37011_16Sgenome     (1449)  CCGAAGCCGGTGGAGTAACCATTTATGGAGCTAGCCGTCGAAGGTGGGAC
                                  1501                                           1550
IPLA37011_Consensus     (1456)  AAATGATTGGGGTGAAGTC-------------------------------
IPLA37011_16Sgenome     (1499)  AAATGATTGGGGTGAAGTCGTAACAAGGTAGCCGTATCGGAAGGTGCGGC
                                  1551             1568
IPLA37011_Consensus     (1475)  ------------------
IPLA37011_16Sgenome     (1549)  TGGATCACCTCCTTTCTA
```

Strain IPLA 37012^T (1465 nucleotides):

Amplicon-derived consensus sequence:

GACCGGTTTGGCTTCCGGGGACTACGGTGCCTAATACATGCAAGTCGAGCGAACGGATAAGGAGCTTGCTCCTTTGAAGTTAGCGGC
GGACGGGTGAGTAACACGTGGGTAACCTACCTATAAGACTGGAATAACTTCGGGAAACCGGAGCTAATGCCGGATAACATTTGGAA
CCGCATGGTTCTAAAGTAAAAGATGGTTTTGCTATCACTTATAGATGGACCCGCGCCGTATTAGCTAGTTGGTAAGGTAACGGCTTAC
CAAGGCAACGATACGTAGCCGACCTGAGAGGGTGATCGGCCACACTGGAACTGAGACACGGTCCAGACTCCTACGGGAGGCAGCA
GTAGGGAATCTTCCGCAATGGACGAAAGTCTGACGGAGCAACGCCGCGTGAGTGATGAAGGTTTTCGGATCGTAAAACTCTGTTATT
AGGGAAGAACAAATGCGTAAGTAACTGTGCGCATCTTGACGGTACCTAATCAGAAAGCCACGGCTAACTACGTGCCAGCAGCCGCG
GTAATACGTAGGTGGCAAGCGTTATCCGGAATTATTGGGCGTAAAGCGCGCGTAGGCGGTTTCTTAAGTCTGATGTGAAAGCCCACG
GCTCAACCGTGGAGGGTCATTGGAAACTGGGAAACTTGAGTACAGAAGAGGAAAGTGGAATTCCATGTGTAGCGGTGAAATGCGC
AGAGATATGGAGGAACACCAGTGGCGAAGGCGACTTTCTGGTCTGTAACTGACGCTGATGTGCGAAAGCGTGGGGATCAAACAGG
ATTAGATACCCTGGTAGTCCACGCCGTAAACGATGAGTGCTAAGTGTTTAGGGGGTTTCCGCCCCTTAGTGCTGCAGCTAACGCATTA
AGCACTCCGCCTGGGGAGTACGACCGCAAGGTTGAAACTCAAAGGAATTGACGGGGACCCGCACAAGCGGTGGAGCATGTGGTTTA
ATTCGAAGCAACGCGAAGAACCTTACCAAATCTTGACATCCTTTGAAAACTCTAGAGATAGAGCCTTCCCCTTCGGGGGACAAAGTG
ACAGGTGGTGCATGGTTGTCGTCAGCTCGTGTCGTGAGATGTTGGGTTAAGTCCCGCAACGAGCGCAACCCTTAAACTTAGTTGCCA
GCATTTAGTTGGGCACTCTAGGTTGACTGCCGGTGACAAACCGGAGGAAGGTGGGGATGACGTCAAATCATCATGCCCCTTATGATT
TGGGCTACACACGTGCTACAATGGACAATACAAAGGGCAGCTAAACCGCGAGGTCATGCAAATCCCATAAAGTTGTTCTCAGTTCGG
ATTGTAGTCTGCAACTCGACTACATGAAGCTGGAATCGCTAGTAATCGTAGATCAGCATGCTACGGTGAATACGTTCCCGGGTCTTGT
ACACACCGCCCGTCACACCACGAGAGTTTGTAACACCCGAAGCCGGTGGAGTAACCATTTATGGAGCTAGC

Alignment amplicon-derived sequence vs WGS data:

```
                                  1                                                50
IPLA37012_Consensus        (1)  ----------------GACCGGTT-----TGGCTTCCGGGGACTACGCTG
IPLA37012_16Sgenome        (1)  TCATAATTCATTTATGGAGAGTTTGATCCTGGCT--CAGGATGAACGCTG
                                  51                                              100
IPLA37012_Consensus       (30)  GCGGCGTGCCTAATACATGCAAGTCGAGCGAACGGATAAGGAGCTTGCTC
IPLA37012_16Sgenome       (49)  GCGGCGTGCCTAATACATGCAAGTCGAGCGAACGGATAAGGAGCTTGCTC
                                  101                                             150
IPLA37012_Consensus       (80)  CTTTGAAGTTAGCGGCGGACGGGTGAGTAACACGTGGGTAACCTACCTAT
IPLA37012_16Sgenome       (99)  CTTTGAAGTTAGCGGCGGACGGGTGAGTAACACGTGGGTAACCTACCTAT
                                  151                                             200
IPLA37012_Consensus      (130)  AAGACTGGAATAACTTCGGGAAACCGGAGCTAATGCCGGATAACATTTGG
IPLA37012_16Sgenome      (149)  AAGACTGGAATAACTTCGGGAAACCGGAGCTAATGCCGGATAACATTTGG
                                  201                                             250
IPLA37012_Consensus      (180)  AACCGCATGGTTCTAAAGTAAAAGATGGTTTTGCTATCACTTATAGATGG
IPLA37012_16Sgenome      (199)  AACCGCATGGTTCTAAAGTAAAAGATGGTTTTGCTATCACTTATAGATGG
                                  251                                             300
IPLA37012_Consensus      (230)  ACCCGCGCCGTATTAGCTAGTTGGTAAGGTAACGGCTTACCAAGGCAACG
IPLA37012_16Sgenome      (249)  ACCCGCGCCGTATTAGCTAGTTGGTAAGGTAACGGCTTACCAAGGCAACG
                                  301                                             350
IPLA37012_Consensus      (280)  ATACGTAGCCGACCTGAGAGGGTGATCGGCCACACTGGAACTGAGACACG
IPLA37012_16Sgenome      (299)  ATACGTAGCCGACCTGAGAGGGTGATCGGCCACACTGGAACTGAGACACG
                                  351                                             400
IPLA37012_Consensus      (330)  GTCCAGACTCCTACGGGAGGCAGCAGTAGGGAATCTTCCGCAATGGACGA
IPLA37012_16Sgenome      (349)  GTCCAGACTCCTACGGGAGGCAGCAGTAGGGAATCTTCCGCAATGGACGA
```

```
                                 401                                              450
IPLA37012_Consensus     (380)   AAGTCTGACGGAGCAACGCCGCGTGAGTGATGAAGGTTTTCGGATCGTAA
IPLA37012_16Sgenome     (399)   AAGTCTGACGGAGCAACGCCGCGTGAGTGATGAAGGTTTTCGGATCGTAA
                                 451                                              500
IPLA37012_Consensus     (430)   AACTCTGTTATTAGGGAAGAACAAATGCGTAAGTAACTGTGCGCATCTTG
IPLA37012_16Sgenome     (449)   AACTCTGTTATTAGGGAAGAACAAATGCGTAAGTAACTGTGCGCATCTTG
                                 501                                              550
IPLA37012_Consensus     (480)   ACGGTACCTAATCAGAAAGCCACGGCTAACTACGTGCCAGCAGCCGCGGT
IPLA37012_16Sgenome     (499)   ACGGTACCTAATCAGAAAGCCACGGCTAACTACGTGCCAGCAGCCGCGGT
                                 551                                              600
IPLA37012_Consensus     (530)   AATACGTAGGTGGCAAGCGTTATCCGGAATTATTGGGCGTAAAGCGCGCG
IPLA37012_16Sgenome     (549)   AATACGTAGGTGGCAAGCGTTATCCGGAATTATTGGGCGTAAAGCGCGCG
                                 601                                              650
IPLA37012_Consensus     (580)   TAGGCGGTTTCTTAAGTCTGATGTGAAAGCCCACGGCTCAACCGTGGAGG
IPLA37012_16Sgenome     (599)   TAGGCGGTTTCTTAAGTCTGATGTGAAAGCCCACGGCTCAACCGTGGAGG
                                 651                                              700
IPLA37012_Consensus     (630)   GTCATTGGAAACTGGGAAACTTGAGTACAGAAGAGGAAAGTGGAATTCCA
IPLA37012_16Sgenome     (649)   GTCATTGGAAACTGGGAAACTTGAGTACAGAAGAGGAAAGTGGAATTCCA
                                 701                                              750
IPLA37012_Consensus     (680)   TGTGTAGCGGTGAAATGCGCAGAGATATGGAGGAACACCAGTGGCGAAGG
IPLA37012_16Sgenome     (699)   TGTGTAGCGGTGAAATGCGCAGAGATATGGAGGAACACCAGTGGCGAAGG
                                 751                                              800
IPLA37012_Consensus     (730)   CGACTTTCTGGTCTGTAACTGACGCTGATGTGCGAAAGCGTGGGGATCAA
IPLA37012_16Sgenome     (749)   CGACTTTCTGGTCTGTAACTGACGCTGATGTGCGAAAGCGTGGGGATCAA
                                 801                                              850
IPLA37012_Consensus     (780)   ACAGGATTAGATACCCTGGTAGTCCACGCCGTAAACGATGAGTGCTAAGT
IPLA37012_16Sgenome     (799)   ACAGGATTAGATACCCTGGTAGTCCACGCCGTAAACGATGAGTGCTAAGT
                                 851                                              900
IPLA37012_Consensus     (830)   GTTTAGGGGGTTTCCGCCCCTTAGTGCTGCAGCTAACGCATTAAGCACTC
IPLA37012_16Sgenome     (849)   GTT-AGGGGGTTTCCGCCCCTTAGTGCTGCAGCTAACGCATTAAGCACTC
                                 901                                              950
IPLA37012_Consensus     (880)   CGCCTGGGGAGTACGACCGCAAGGTTGAAACTCAAAGGAATTGACGGGGA
IPLA37012_16Sgenome     (898)   CGCCTGGGGAGTACGACCGCAAGGTTGAAACTCAAAGGAATTGACGGGGA
                                 951                                             1000
IPLA37012_Consensus     (930)   CCCGCACAAGCGGTGGAGCATGTGGTTTAATTCGAAGCAACGCGAAGAAC
IPLA37012_16Sgenome     (948)   CCCGCACAAGCGGTGGAGCATGTGGTTTAATTCGAAGCAACGCGAAGAAC
                                 1001                                             1050
IPLA37012_Consensus     (980)   CTTACCAAATCTTGACATCCTTTGAAAACTCTAGAGATAGAGCCTTCCCC
IPLA37012_16Sgenome     (998)   CTTACCAAATCTTGACATCCTTTGAAAACTCTAGAGATAGAGCCTTCCCC
                                 1051                                             1100
IPLA37012_Consensus    (1030)   TTCGGGGGACAAAGTGACAGGTGGTGCATGGTTGTCGTCAGCTCGTGTCG
IPLA37012_16Sgenome    (1048)   TTCGGGGGACAAAGTGACAGGTGGTGCATGGTTGTCGTCAGCTCGTGTCG
                                 1101                                             1150
IPLA37012_Consensus    (1080)   TGAGATGTTGGGTTAAGTCCCGCAACGAGCGCAACCCTTAAACTTAGTTG
IPLA37012_16Sgenome    (1098)   TGAGATGTTGGGTTAAGTCCCGCAACGAGCGCAACCCTTAAACTTAGTTG
                                 1151                                             1200
IPLA37012_Consensus    (1130)   CCAGCATTTAGTTGGGCACTCTAGGTTGACTGCCGGTGACAAACCGGAGG
IPLA37012_16Sgenome    (1148)   CCAGCATTTAGTTGGGCACTCTAGGTTGACTGCCGGTGACAAACCGGAGG
                                 1201                                             1250
IPLA37012_Consensus    (1180)   AAGGTGGGGATGACGTCAAATCATCATGCCCCTTATGATTTGGGCTACAC
IPLA37012_16Sgenome    (1198)   AAGGTGGGGATGACGTCAAATCATCATGCCCCTTATGATTTGGGCTACAC
                                 1251                                             1300
IPLA37012_Consensus    (1230)   ACGTGCTACAATGGACAATACAAAGGGCAGCTAAACCGCGAGGTCATGCA
IPLA37012_16Sgenome    (1248)   ACGTGCTACAATGGACAATACAAAGGGCAGCTAAACCGCGAGGTCATGCA
                                 1301                                             1350
IPLA37012_Consensus    (1280)   AATCCCATAAAGTTGTTCTCAGTTCGGATTGTAGTCTGCAACTCGACTAC
IPLA37012_16Sgenome    (1298)   AATCCCATAAAGTTGTTCTCAGTTCGGATTGTAGTCTGCAACTCGACTAC
                                 1351                                             1400
IPLA37012_Consensus    (1330)   ATGAAGCTGGAATCGCTAGTAATCGTAGATCAGCATGCTACGGTGAATAC
IPLA37012_16Sgenome    (1348)   ATGAAGCTGGAATCGCTAGTAATCGTAGATCAGCATGCTACGGTGAATAC
                                 1401                                             1450
IPLA37012_Consensus    (1380)   GTTCCCGGGTCTTGTACACACCGCCCGTCACACCACGAGAGTTTGTAACA
IPLA37012_16Sgenome    (1398)   GTTCCCGGGTCTTGTACACACCGCCCGTCACACCACGAGAGTTTGTAACA
```

```
                                       1451                                              1500
IPLA37012_Consensus    (1430) CCCGAAGCCGGTGGAGTAACCATTTATGGAGCTAGC--------------
IPLA37012_16Sgenome    (1448) CCCGAAGCCGGTGGAGTAACCATTTATGGAGCTAGCCGTCGAAGGTGGGA
                                       1501                                              1550
IPLA37012_Consensus    (1466) --------------------------------------------------
IPLA37012_16Sgenome    (1498) CAAATGATTGGGGTGAAGTCGTAACAAGGTAGCCGTATCGGAAGGTGCGG
                                       1551               1569
IPLA37012_Consensus    (1466) -------------------
IPLA37012_16Sgenome    (1548) CTGGATCACCTCCTTTCTA
```

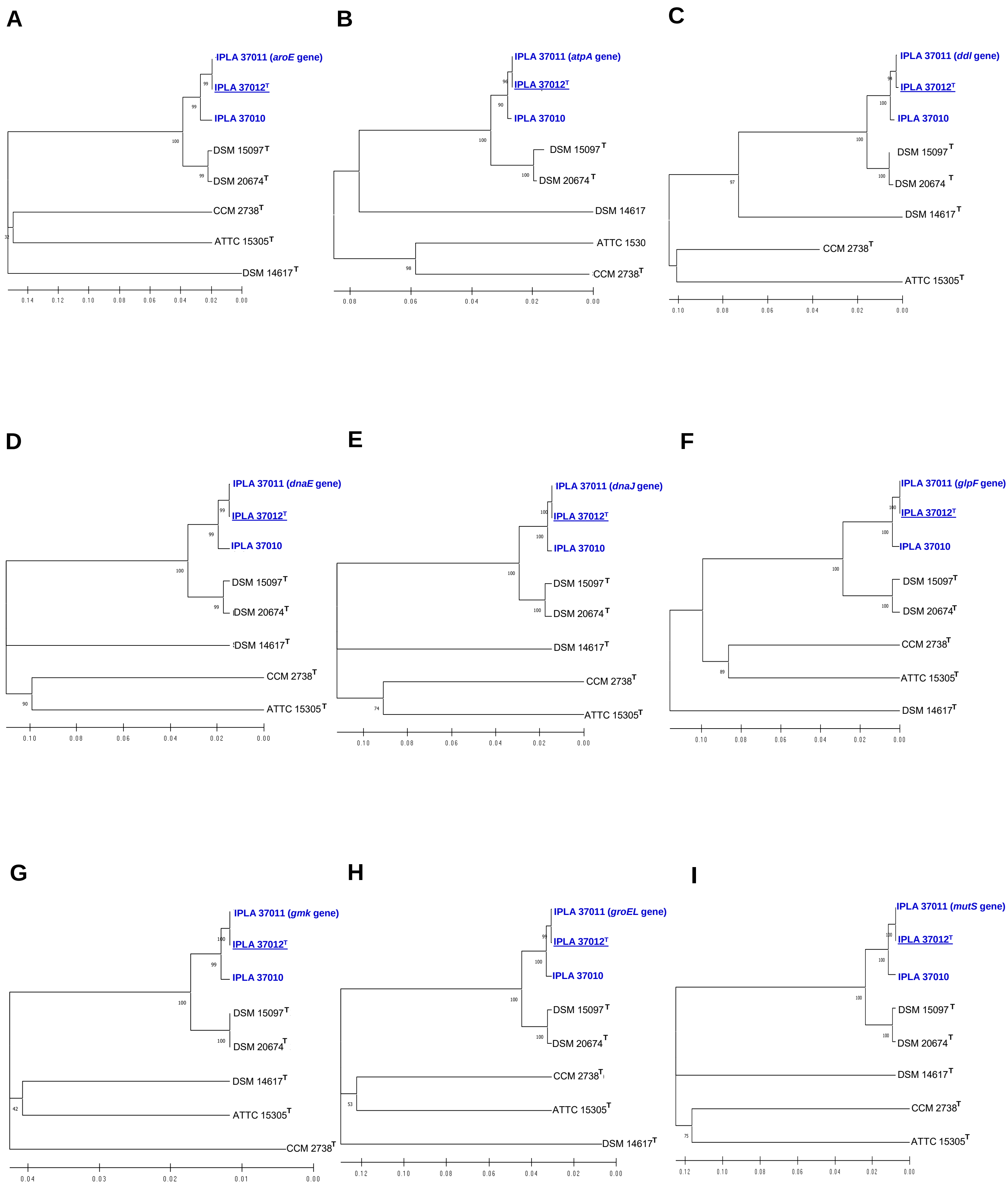
A
IPLA 37011 (aroE gene)
IPLA 37012T
IPLA 37010
DSM 15097T
DSM 20674T
CCM 2738T
ATTC 15305T
DSM 14617T
0.14 0.12 0.10 0.08 0.06 0.04 0.02 0.00
B
IPLA 37011 (atpA gene)
IPLA 37012T
IPLA 37010
DSM 15097T
DSM 20674T
DSM 14617
ATTC 1530
CCM 2738T
0.08 0.06 0.04 0.02 0.00
C
IPLA 37011 (ddl gene)
IPLA 37012T
IPLA 37010
DSM 15097T
DSM 20674T
DSM 14617T
CCM 2738T
ATTC 15305T
0.10 0.08 0.06 0.04 0.02 0.00
D
IPLA 37011 (dnaE gene)
IPLA 37012T
IPLA 37010
DSM 15097T
DSM 20674T
DSM 14617T
CCM 2738T
ATTC 15305T
0.10 0.08 0.06 0.04 0.02 0.00
E
IPLA 37011 (dnaJ gene)
IPLA 37012T
IPLA 37010
DSM 15097T
DSM 20674T
DSM 14617T
CCM 2738T
ATTC 15305T
0.10 0.08 0.06 0.04 0.02 0.00
F
IPLA 37011 (glpF gene)
IPLA 37012T
IPLA 37010
DSM 15097T
DSM 20674T
CCM 2738T
ATTC 15305T
DSM 14617T
0.10 0.08 0.06 0.04 0.02 0.00
G
IPLA 37011 (gmk gene)
IPLA 37012T
IPLA 37010
DSM 15097T
DSM 20674T
DSM 14617T
ATTC 15305T
CCM 2738T
0.04 0.03 0.02 0.01 0.00
H
IPLA 37011 (groEL gene)
IPLA 37012T
IPLA 37010
DSM 15097T
DSM 20674T
CCM 2738T
ATTC 15305T
DSM 14617T
0.12 0.10 0.08 0.06 0.04 0.02 0.00
I
IPLA 37011 (mutS gene)
IPLA 37012T
IPLA 37010
DSM 15097T
DSM 20674T
DSM 14617T
CCM 2738T
ATTC 15305T
0.12 0.10 0.08 0.06 0.04 0.02 0.00

J

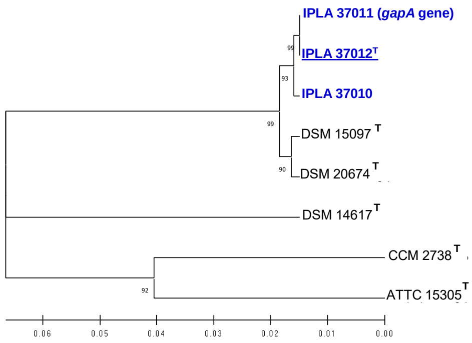

IPLA 37011 (gapA gene)
IPLA 37012T
IPLA 37010
DSM 15097 T
DSM 20674 T
DSM 14617T
CCM 2738 T
ATTC 15305T
0.06
0.05
0.04
0.03
0.02
0.01
0.00

K

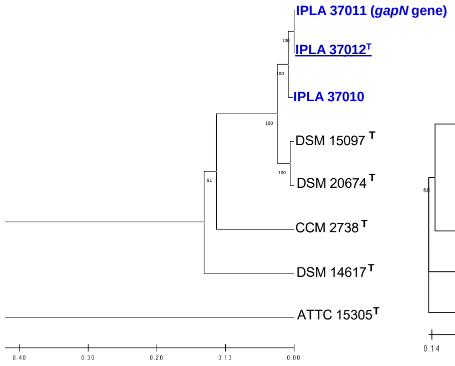

IPLA 37011 (gapN gene)
IPLA 37012T
IPLA 37010
DSM 15097 T
DSM 20674 T
CCM 2738 T
DSM 14617T
ATTC 15305T
0.40
0.30
0.20
0.10
0.00

L

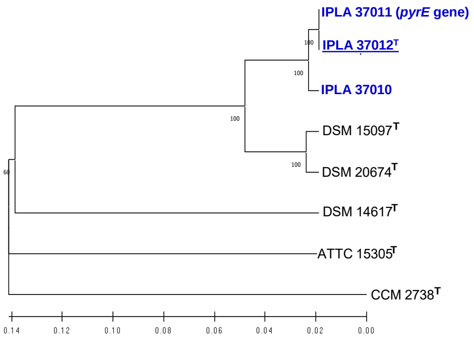

IPLA 37011 (pyrE gene)
IPLA 37012T
IPLA 37010
DSM 15097T
DSM 20674T
DSM 14617T
ATTC 15305T
CCM 2738T
0.14
0.12
0.10
0.08
0.06
0.04
0.02
0.00

M

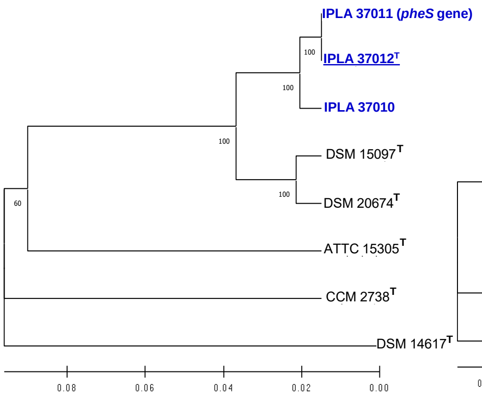

IPLA 37011 (pheS gene)
IPLA 37012T
IPLA 37010
DSM 15097T
DSM 20674T
ATTC 15305T
CCM 2738T
DSM 14617T
0.08
0.06
0.04
0.02
0.00

N

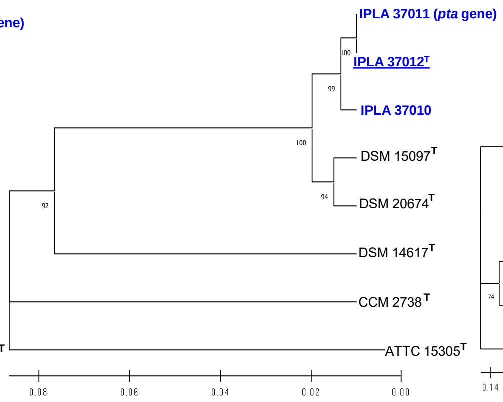

IPLA 37011 (pta gene)
IPLA 37012T
IPLA 37010
DSM 15097T
DSM 20674T
DSM 14617T
CCM 2738 T
ATTC 15305T
0.08
0.06
0.04
0.02
0.00

O

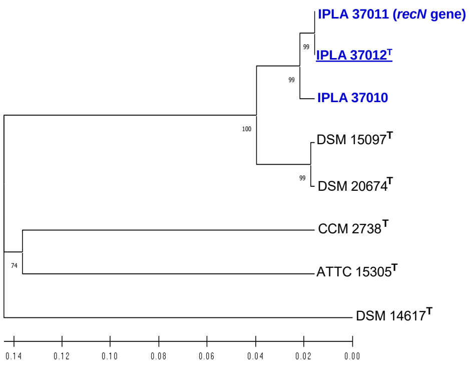

IPLA 37011 (recN gene)
IPLA 37012T
IPLA 37010
DSM 15097T
DSM 20674T
CCM 2738T
ATTC 15305T
DSM 14617T
0.14
0.12
0.10
0.08
0.06
0.04
0.02
0.00

P

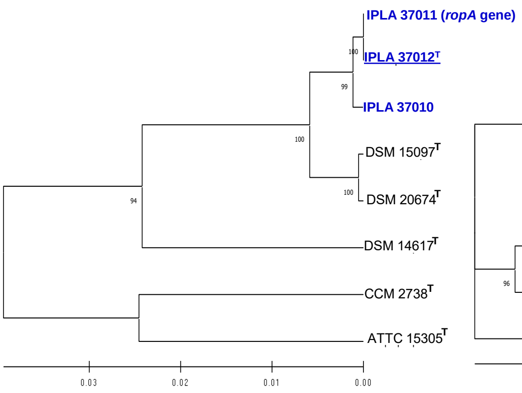

IPLA 37011 (ropA gene)
IPLA 37012T
IPLA 37010
DSM 15097T
DSM 20674T
DSM 14617T
CCM 2738T
ATTC 15305T
0.03
0.02
0.01
0.00

Q

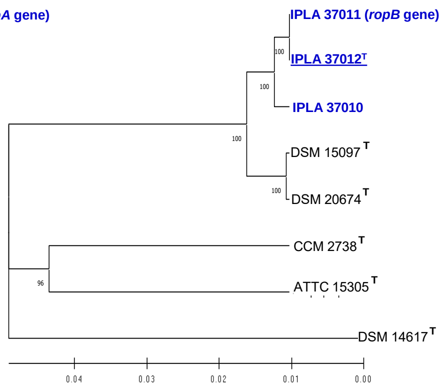

IPLA 37011 (ropB gene)
IPLA 37012T
IPLA 37010
DSM 15097 T
DSM 20674 T
CCM 2738 T
ATTC 15305 T
DSM 14617 T
0.04
0.03
0.02
0.01
0.00

R

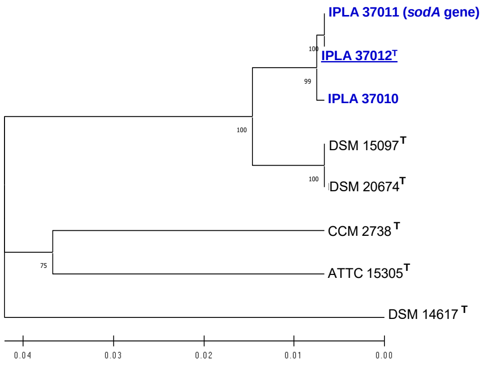

IPLA 37011 (sodA gene)
IPLA 37012T
IPLA 37010
DSM 15097T
DSM 20674T
CCM 2738 T
ATTC 15305T
DSM 14617 T
0.04
0.03
0.02
0.01
0.00

S

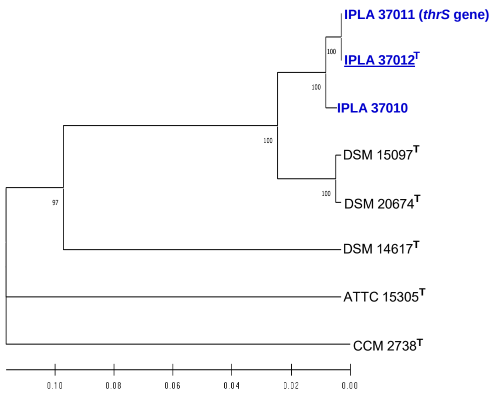

T

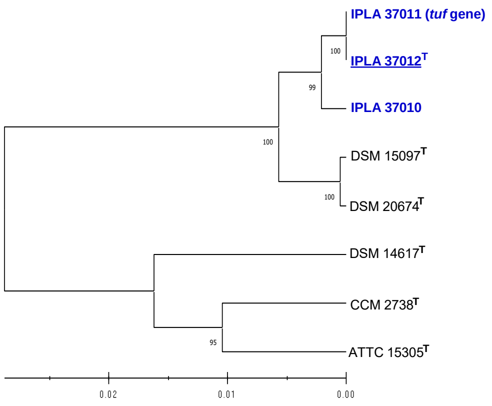

Supplementary Figure 3.- Individual phylogenetic trees based on gene sequences extracted from whole genome sequencing data inferred by using the maximum likelihood method and the General Time Reversible model. The percentage of 1,000 trees in which the associated taxa clustered together is shown below the branches. Trees are drawn to scale, with branch lengths measured in the number of substitutions per site. Key of the genes from A to T: *aroE, atpA, ddlA, dnaE, dnaJ, glpF, Gmk, groEL, mutS, gapA, gapN, pyrE, pheS, Pta, recN, rpoA, rpoB, sodA, thrS*, and *tuf*.

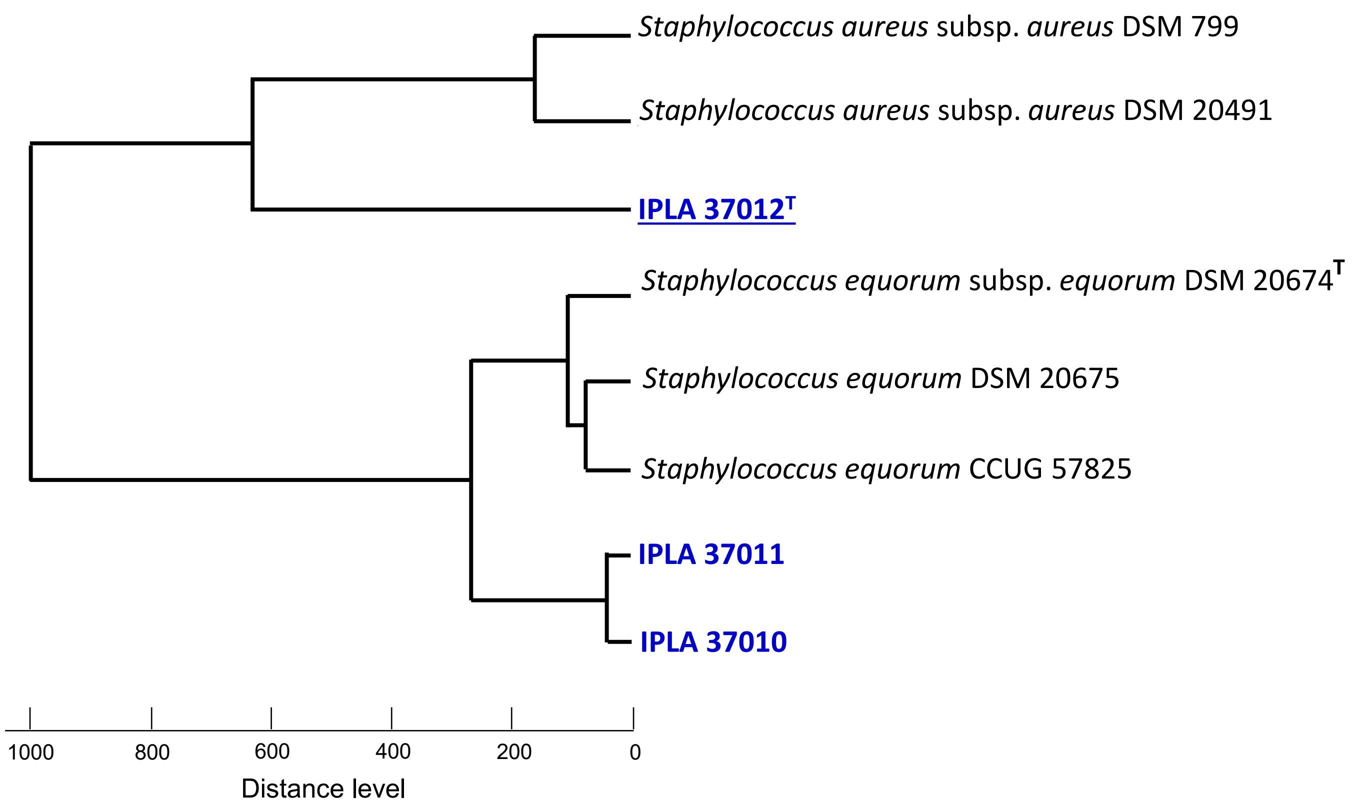

Supplementary Figure 4.- Dendrogram of similarity obtained by cluster analysis of MALDI-TOF MS spectra of the three *S. caseorum* strains of this study from cheese and strains from the DSMZ database sowing the closest similarity. The distance level is displayed in relative units.
